# Supplementary material for: Valorization of Pomegranate Peel: Mechanisms and Clinical Applications in Irritable Bowel Syndrome Management
Source: Int J Mol Sci. 2025 Apr 9;26(8):3530. doi: 10.3390/ijms26083530 (PMC12026873; doi:10.3390/ijms26083530)
Supplement: Supplementary file 1 [file ijms-26-03530-s001.zip › ijms-3532366-supplementary.pdf]

Supplementary Materials

Valorization of Pomegranate Peel: Mechanisms and Clinical Applications in Irritable Bowel Syndrome Management

Table S1. The 46 candidate compounds in PP.

No.	Name	CAS
1	β-Glucogalin	13405-60-2
2	Gallic acid	149-91-7
3	Punicalin	65995-64-4
4	2-O-Galloylpunicalin	103488-45-5
5	Pedunculagin	7045-42-3
6	Urolithin D	131086-98-1
7	Gallocatechin	970-73-0
8	Punicalagin	65995-63-3
9	(-)-Epigallocatechin	970-74-1
10	sanguisorbic acid dilactone	82203-11-0
11	Procyanidin B2	15514-06-4
12	Tellimagrandin I	79786-08-6
13	Urolithin A	1143-70-0
14	Granatin A	161205-11-4
15	Methyl gallate	99-24-1
16	Catechin	154-23-4
17	Casuarinin	79786-01-9
18	Corilagin	23094-69-1
19	Castalin	19086-75-0
20	(-)-Gallocatechin gallate	4233-96-9
21	Granatin B	77322-54-4
22	Valoneic acid dilactone	60202-70-2
23	Ellagic acid	476-66-4
24	kaempferol-3-O-β-D-glucopyranoside	480-10-4
25	3-Glucosylquercetin	482-35-9
26	kaempferol-3-O-β-d-xylopyranoside	61117-16-6
27	5-hydroxymethylfurfural	67-47-0
28	Pelargonidin	7690-51-9
29	3,3'-Di-O-methylellagic acid 4'-glucoside	51803-68-0
30	Apigenin	520-36-5
31	monoaryl glycosides β-hydroxypropiovanillone 3-O-β-D-glucopyranoside	/
32	3-O-(β-D-glucopyranosyl)-1-(3′,5′-dimethoxy-4′-hydr-oxyphenyl)-1-propanone	/
33	5-hydroxymethylfuran-3-carboxylic acid	246178-75-6
34	(1′R, 3′S, 5′R, 8′S, 2Z, 4E)-dihydrophaseic acid 3′-O-β-d-glucopyranoside	/
35	Cyanidin	13306-05-3
36	Pelletierine	2858-66-4

37	Isopelletierine	4396-01-4
38	pseudopelletierine	552-70-5
39	Oleanic Acid	508-02-1
40	kaempferol	520-18-3
41	β-Sitosterol	83-46-5
42	luteolin	491-70-3
43	quercetin	117-39-5
44	gallocatechin-(4-8)-catechin	/
45	gallocatechin-(4-8)-gallocatechin	/
46	catechin-(4-8)-gallocatechin	/

Table S2. The references of the 39 compounds.

No.	Name	CAS	Class	References	No.	Name	CAS	Class	References
PP 01	β-Glucogalin	13405-60-2	Polyphenols	[1]	PP 21	Granatin B	77322-54-4	Polyphenols	[5]
PP 02	Gallic acid	149-91-7	Polyphenols	[2]	PP 22	Valoneic acid dilactone	60202-70-2	Polyphenols	[8]
PP 03	Punicalin	65995-64-4	Polyphenols	[3]	PP 23	Ellagic acid	476-66-4	Polyphenols	[5]
PP 04	2-O-Galloylpunicalin	103488-45-5	Polyphenols	[4]	PP 24	Kaempferol-3-O-β-D-glucopyranoside	480-10-4	Polyphenols	[13]
PP 05	Pedunculagin	7045-42-3	Polyphenols	[5]	PP 25	3-Glucosylquercetin	482-35-9	Polyphenols	[14]
PP 06	Urolithin D	131086-98-1	Polyphenols	[6]	PP 26	5-Hydroxymethylfurfural	67-47-0	Furfurals	[15]
PP 07	Gallocatechin	970-73-0	Polyphenols	[5]	PP 27	Pelargonidin	7690-51-9	Polyphenols	[16]
PP 08	Punicalagin	65995-63-3	Polyphenols	[5]	PP 28	3,3'-Di-O-methylellagic acid 4'-glucoside	51803-68-0	Polyphenols	[17]
PP 09	(-)-Epigallocatechin	970-74-1	Polyphenols	[7]	PP 29	Apigenin	520-36-5	Polyphenols	[16]
PP 10	Sanguisorbic acid dilactone	82203-11-0	Polyphenols	[8]	PP 30	5-Hydroxymethylfurfural	67-47-0	Furfurals	[18]

PP 11	Procyanidin B2	15514-06-4	Polyphenols	[9]	PP 31	Cyanidin	13306-05-3	Polyphenols	[16]
PP 12	Tellimagrandin I	79786-08-6	Polyphenols	[5]	PP 32	Pelletierine	2858-66-4	Alkaloids	[19]
PP 13	Urolithin A	1143-70-0	Polyphenols	[6]	PP 33	Isopelletierine	4396-1-4	Alkaloids	[20]
PP 14	Granatin A	161205-11-4	Polyphenols	[5]	PP 34	Pseudopelletierine	552-70-5	Alkaloids	[19]
PP 15	Methyl gallate	99-24-1	Polyphenols	[10]	PP 35	Oleanic Acid	508-02-1	Triterpenoids	[21]
PP 16	Catechin	154-23-4	Polyphenols	[5]	PP 36	Kaempferol	520-18-3	Polyphenols	[5]
PP 17	Casuarinin	79786-01-9	Polyphenols	[5]	PP 37	β-Sitosterol	83-46-5	Triterpenoids	[22]
PP 18	Corilagin	23094-69-1	Polyphenols	[5]	PP 38	Luteolin	491-70-3	Polyphenols	[5]
PP 19	Castalin	19086-75-0	Polyphenols	[11]	PP 39	Quercetin	117-39-5	Polyphenols	[16]
PP 20	(-)-Gallocatechin gallate	4233-96-9	Polyphenols	[12]					

References

[1] Silva L. D. O., Garrett R., Monteiro M. L. G., Conte-Junior C. A., Torres A. G. Pomegranate (Punica granatum) peel fractions obtained by supercritical CO2 increase oxidative and colour stability of bluefish (Pomatomus saltatrix) patties treated by UV-C irradiation[J]. *Food Chemistry*, **2021**, *362*: 130159. doi.org/10.1016/j.foodchem.2021.130159

[2] Sood A., Gupta M. Extraction process optimization for bioactive compounds in pomegranate peel[J]. *Food Bioscience*, **2015**, *12*: 100-106. doi.org/10.1016/j.fbio.2015.09.004

[3] Glazer I., Masaphy S., Marciano P., Bar-Ilan I., Holland D., Kerem Z., Amir R. Partial identification of antifungal compounds from Punica granatum peel extracts[J]. *Journal of agricultural and food chemistry*, **2012**, *60*(19): 4841-4848. doi.org/10.1021/jf300330y

[4] Hernández-Corroto E., Marina M. L., García M. C. Extraction and identification by high resolution mass spectrometry of bioactive substances in different extracts obtained from pomegranate peel[J]. *Journal of Chromatography A*, **2019**, *1594*: 82-92. doi.org/10.1016/j.chroma.2019.02.018

[5] Akhtar S., Ismail T., Fraternale D., Sestili P. Pomegranate peel and peel extracts: Chemistry and food features[J]. *Food chemistry*, **2015**, *174*: 417-425. doi.org/10.1016/j.foodchem.2014.11.035

[6] Xiang Q., Li M., Wen J., Ren F., Yang Z., Jiang X., Chen Y. The bioactivity and applications of pomegranate peel extract: A review[J]. *Journal of Food Biochemistry*, **2022**, *46*(7): e14105. doi.org/10.1111/jfbc.14105

[7] Peršurić Ž., Saftić Martinović L., Malenica M., Gobin I., Pedisić S., Dragović-Uzelac V., Kraljević Pavelić S. Assessment of the biological activity and phenolic composition of ethanol extracts of pomegranate (Punica granatum L.) peels[J]. *Molecules*, **2020**, *25*(24): 5916. doi.org/10.3390/molecules25245916

[8]Garcia-Villalba R., Espín J.C., Aaby K., Alasalvar C., Heinonen M., Jacobs G., Voorspoels S., Koivumaki T., Kroon P.A., Pelvan E. Saha S. Validated method for the characterization and quantification of extractable and nonextractable ellagitannins after acid hydrolysis in pomegranate fruits, juices, and extracts[J]. *Journal of Agricultural and Food Chemistry*, **2015**, *63*(29): 6555-6566. doi.org/10.1021/acs.jafc.5b02062

[9] Marra F., Petrovicova B., Canino F., Maffia A., Mallamaci C., Muscolo A. Pomegranate wastes are rich in bioactive compounds with potential benefit on human health[J]. *Molecules*, **2022**, *27*(17), 5555. doi.org/10.3390/molecules27175555

[10] González‐Hidalgo I., Bañón S., Ros J. M. Evaluation of table olive by‐product as a source of natural antioxidants[J]. *International Journal of Food Science and Technology*, **2012**, *47*(4), 674-681. doi:10.1111/j.1365-2621.2011.02892.x

[11] Elwej A., Grojja Y., Ghorbel I., Boudawara O., Jarraya R., Boudawara T., Zeghal N. Barium chloride induces redox status unbalance, upregulates cytokine genes expression and confers hepatotoxicity in rats—alleviation by pomegranate peel[J]. *Environmental Science and Pollution Research*, **2016**, *23*: 7559-7571. doi.org/10.1007/s11356-015-6023-0

[12] Díaz-Mula H. M., Tomás-Barberán F. A., García-Villalba R. Pomegranate fruit and juice (cv. Mollar), rich in ellagitannins and anthocyanins, also provide a significant content of a wide range of proanthocyanidins[J]. *Journal of Agricultural and Food Chemistry*, **2019**, *67*(33): 9160-9167. doi.org/10.1021/acs.jafc.8b07155

[13] Ambigaipalan P., de Camargo A. C., Shahidi F. Phenolic compounds of pomegranate byproducts (outer skin, mesocarp, divider membrane) and their antioxidant activities[J]. *Journal of Agricultural and Food Chemistry*, **2016**, *64*(34): 6584-6604. doi.org/10.1021/acs.jafc.6b02950

[14] El-Hadary A. E., Ramadan, M. F. Phenolic profiles, antihyperglycemic, antihyperlipidemic, and antioxidant properties of pomegranate (Punica granatum) peel extract[J]. *Journal of Food Biochemistry*, **2019**, *43*(4): e12803. doi.org/10.1111/jfbc.12803

[15] Ravikumar K. V. G., Sudakaran S. V., Ravichandran K., Pulimi M., Natarajan C., Mukherjee A. Green synthesis of NiFe nano particles using Punica granatum peel extract for tetracycline removal[J]. *Journal of Cleaner Production*, **2019**, *210*: 767-776. doi.org/10.1016/j.jclepro.2018.11.108

[16] Singh B., Singh J. P., Kaur A., Singh N. Phenolic compounds as beneficial phytochemicals in pomegranate (Punica granatum L.) peel: A review[J]. *Food Chemistry*, **2018**, *261*: 75-86. doi.org/10.1016/j.foodchem.2018.04.039

[17] Yuan T., Ma H., Liu W., Niesen D.B., Shah N., Crews R., Rose K.N., Vattem D.A. Seeram N.P. Pomegranate's neuroprotective effects against Alzheimer's disease are mediated by urolithins, its ellagitannin-gut microbial derived metabolites[J]. *ACS Chemical Neuroscience*, **2016**, *7*(1):26-33. doi.org/10.1021/acschemneuro.5b00260.

[18] Kaur R., Kaushal S., Sharma P. Antimicrobial and antioxidant potential of pomegranate (Punica granatum L.) peel[J]. *Int. J. Chem. Stud*, **2018**, *6*: 3441-3449.

[19] Neuhöfer H., Witte L., Gorunovic M., Czygan F. C. Alkaloids in the bark of Punica granatum L.(pomegranate) from Yugoslavia[J]. *Pharmazie*, **1993**, *48*(5): 389-391.

[20] Keogh M. F., O'Donovan D. G. Biosynthesis of some alkaloids of Punica granatum and Withania somnifera[J]. *Journal of the Chemical Society C: Organic*, **1970**, *13*:1792-1797. doi.org/10.1039/J39700001792

[21] Ding W., Wang H., Zhou Q., Wu C., Gao X., Cheng X., Tian L., Wang C. Simultaneous determination of polyphenols and triterpenes in pomegranate peel based on high - performance liquid chromatography fingerprint by solvent extraction and ratio blending method in tandem with wavelength switching[J]. *Biomedical Chromatography*, **2019**, *33*(12): e4690. doi.org/10.1002/bmc.4690

[22] Sun S., Huang S., Shi Y., Shao Y., Qiu J., Sedjoah R.C.A.A., Yan Z., Ding L., Zou D., Xin Z. Extraction, isolation, characterization and antimicrobial activities of non-extractable polyphenols from pomegranate peel[J]. *Food Chemistry*, **2021**, *351*: 129232. doi.org/10.1016/j.foodchem.2021.129232

Table S3. All predicted targets associated with potentially active compounds.

No.	Compound	Target
PP01	β-Glucogalin	APEX1;TDP1;BLM;TTR;NFKB1;DUSP3;CTSD;RPS6KA3;TLR4;GPR35;PLAT;NFE2L2;NR1A1;HSP90AA1;CHRM3;DNMT1;NTRK3;GPR6;SLC9A1;QRFPR;NR3C2;TRIM24;ABCC1;GLRA1;GPR17;HDAC5;MAOA;PDGFRA;METAP2;STAT1;F13A1;PLAU;NPC1;MIF;PRCP;CYP3A4;CDK5;RORB;CHRM2;CBX4;CFTR;CHRM1;RXFP1;GRK5;GUSB;TFPI;ACACA;MAP2K2;AXL;PRSS1;SCD;CCR2;TDO2;GRB2;ACACB
PP02	Gallic acid	FUT7;LMNA;GALNT2;APEX1;TTR;TDP1;TRIM24;NPC1;CDK5;GLRA1;RAB9A;NR1A1;MDM4;NR4A1;GPR35;RORB;CYP3A4;STAT1;CTSD;RPS6KA3;CHRM5;LGALS3;GUSB;NFKB1;BLM;MAP2K2;QRFPR;NFE2L2;IRAK1;PLAT;SLC9A1;PSMB9;EGLN1;TBXAS1;GPR6;CHRM3;PIN1;AKR1C2;CHRM4;DUSP3;NOX1;TLR4;ULK3;ERAP1;ERN1;ACACA;F13A1;CASP6;PRCP;RPS6KA1;EPHB2;KDM4A;NTRK3;NR3C2;ACVR1B;AVPR1B;CHRM1;ADRB1;CHRM2;EPHB3;HDAC5;ABCC1;PDGFRA;MAOA

PP03	Punicalin	TDP1;POLB;RECQL;USP2;BLM;APEX1;MAOA;TRIM24;DUSP3;CTSD;HSP90AA1;PTGS2;NTRK3;NR1A1;NR3C2;GPR35;TTR;GLRA1;RPS6KA1;TMEM173;TDO2;CDK5;GLS;F13A1;NOS2;GPR17;PIN1;CLK4;ACACA;GSK3A;GRK5;BMP2K;CYP3A4;NFE2L2;PRCP;NOTUM;HSP90AB1;MAP2K2;RPS6KA3;ZAP70;TLR4;F2R;TLR8;MINK1;SLC9A1;P2RX4;GPR6;KEAP1;METAP2;LDHB;CLK2;ACVRL1;PSMB9;AKR1C1;TFPI;ACACB;GPBAR1;CFTR;MARK4;HDAC8;HDAC10;FCGRT;ABCC1;GRB2;MAP3K11;LGALS3;EGLN1;QRFPR;HDAC5;CETP;ITK;ACVR1B;CTSS;NR4A1;PDGFRA;SCD;NT5E
PP04	2-O-Galloylpunicalin	TDP1;APEX1;MAOA;DUSP3;TRIM24;TTR;CTSD;BLM;GPR35;NTRK3;HSP90AA1;NR3C2;CYP3A4;TLR4;CDK5;NR1A1;GLS;RPS6KA3;NOS2;GLRA1;GPR17;F13A1;RPS6KA1;NFE2L2;ACACA;PDGFRA;PIN1;GSK3A;GPR6;METAP2;PTGS2;GRK5;SLC9A1;TDO2;DNMT1;PRCP;CFTR;NPC1;STAT1;KEAP1;TMEM173;MAP2K2;HDAC5;TFPI;PRSS1;HSP90AB1;ACVRL1;IMPDH2;RORB;CLK4;PLAT;ACACB;BRD2;P2RX7;NOTUM;QRFPR;GRB2;RXFP1;LDHB;LYN;P2RX4;HDAC8;PSMB9;NT5E
PP05	Pedunculagin	F10;APEX1;TDP1;MAOA;TRIM24;NR1A1;DUSP3;CTSD;NR3C2;PTGS2;EGLN1;GLRA1;F13A1;NTRK3;PIN1;TMEM173;GPR17;TTR;TDO2;CDK5;GLS;NFKB1;HSP90AA1;CYP3A4;PSMB9;RORB;ABCC1;SLC9A1;NOS2;TLR4;KEAP1;STAT3;ACACA;GPR6;PDGFRA;RPS6KA1;PRCP;METAP2;BMP2K;GPR55;IMPDH2;TFPI;CLK4;NFE2L2;QRFPR;RPS6KA3;CFTR;F2R;TMPRSS6;GPR35;ZAP70;ACVRL1;P2RX4;LGALS3;NOTUM;ACACB;TLR8;MARK4;MINK1;FPRL2;LDHB;TYRO3;NR4A1;SCD;GPBAR1;MAP2K2;HDAC2;CETP
PP06	Urolithin D	APEX1;TDP1;DUSP3;CTSD;GPR35;CLK4;TTR;HSP90AA1;RPS6KA3;BLM;NR1A1;TRIM24;GPR6;NTRK3;MAOA;CYP3A4;MAP2K2;METAP2;F13A1;TLR4;NR3C2;PIN1;DNMT1;GPR17;AKR1C1;GRK5;NFE2L2;PRCP;NOS2;QRFPR;NR4A1;P2RX4;BMP2K;CPT1B;NPC1;NFKB1;CDK5;PLAT;SCD;RORB;TFPI;P2RX7;GLRA1;ACACA;CFTR;GLS;ZAP70;PDGFRA;ABCC1;RXFP1;TYRO3;FCGRT;ERN1;SLC9A1;GPBAR1;TDO2;HDAC10;GUSB;CCR1;CCR2;ADRB1;ERAP1;FLT1;RPS6KA1
PP07	Gallocatechin	APEX1;TDP1;NFKB1;MAOA;TTR;TRIM24;NR1A1;ABCC1;EGLN1;CLK4;RPS6KA1;RPS6KA3;NR3C2;CTSD;DUSP3;NTRK3;OPRM1;GPR17;F13A1;GLRA1;ROCK1;GRM5;PRCP;BLM;QRFPR;SLC9A1;DNMT1;GPR55;RORB;LGALS3;MARK4;LYN;IL23R;KEAP1;CDK5;STAT1;RPS6KA6;TMEM173;CETP;ACVRL1;MAP2K2;CASP8;TFPI;ZAP70;CHRM5;TLR8;HDAC5;FCGRT;FFAR2;PROC;ACACA;BRAF;SCD;ITK;ACE;ERAP1;F2R;PSMB9;ACVR1B;FPRL2;PIN1;GRB2;PDGFRA;DRD3;HDAC11;KDM6B
PP08	Punicalagin	BACE1;APEX1;TDP1;MAOA;TRIM24;DUSP3;PTGS2;CTSD;NR1A1;GPR35;NR3C2;NTRK3;CDK5;PIN1;HSP90AA1;TTR;GLS;ACACA;GLRA1;F13A1;GPR17;RPS6KA1;NOS2;TMEM173;TDO2;CLK4;CYP3A4;BMP2K;NOTUM;GSK3A;PRCP;NFE2L2;ZAP70;GPR6;TLR4;SLC9A1;P2RX4;GPR55;TFPI;PSMB9;CFTR;ERN1;METAP2;GPBAR1;KEAP1;MAP2K2;F2R;HDAC8;LDHB;ACACB;ACVRL1;LGALS3;GRK5;AKR1C1;EGLN1;MINK1;EIF2AK1;CLK2;RPS6KA3;RORB;FPRL2;STAT3;MARK4;HDAC2;BLM;PDGFRA;ITK;NR4A1;P2RX7;HDAC10SCD
PP09	(-)-Epigallocatechin	APEX1;TDP1;NFKB1;MAOA;TTR;TRIM24;NR1A1;ABCC1;EGLN1;CLK4;RPS6KA1;RPS6KA3;NR3C2;CTSD;DUSP3;NTRK3;OPRM1;GPR17;F13A1;GLRA1;ROCK1;GRM5;PRCP;BLM;QRFPR;SLC9A1;DNMT1;GPR55;RORB;LGALS3;MARK4;LYN;IL23R;KEAP1;CDK5;STAT1;RPS6KA6;TMEM173;CETP;ACVRL1;MAP2K2;CASP8;TFPI;ZAP70;CHRM5;TLR8;HDAC5;FCGRT;FFAR2;PROC;ACACA;BRAF;SCD;ITK;ACE;ERAP1;F2R;PSMB9;ACVR1B;FPRL2;PIN1;GRB2;PDGFRA;DRD3;HDAC11;KDM6B
PP10	sanguisorbic acid dilactone	APEX1;TTR;TRIM24;TDP1;DUSP3;GPR35;NPC1;NR1A1;CYP3A4;RORB;GLRA1;ADRB1;NR4A1;MDM4;RAB9A;MIF;HSP90AA1;MAOA;CTSD;ERN1;F13A1;CLK4;NFE2L2;GUSB;ACACA;LGALS3;QRFPR;CCR1;CDK5;MAP2K2;NTRK3;CHRM5;GPR17;HDAC2;PLAT;PRCP;TLR4;CHRM1;ERAP1;TBXAS1;PRSS1;NR3C2;IMPDH2;AXL;TBXA2R;AKR1C1;CFTR;NOX1;CASP6;P2RX4;PSMB9;EGLN1;RPS6KA3;AVPR1B;CBX4;ACACB;RXFP1;FLT1

PP11	Procyanidin B2	TDP1;APEX1;MAOA;NFKB1;NR1A1;TRIM24;TTR;CLK4;ABCC1;NTRK3;OPRM1;CTSD;RPS6KA1;NR3C2;PRCP;EGLN1;HDAC5;GLRA1;TMEM173;F13A1;DUSP3;CETP;BLM;GRM5;LGALS3;GUSB;PROC;ZAP70;KEAP1;TFPI;ACVRL1;GPR17;SLC9A1;GPBAR1;CHRM5;BRD4;GLS;FCGRT;RPS6KA6;ACACA;ERAP1;METAP2;HDAC11;SCD;ITK;STAT1;NOS2;CYSLTR2;QRFPR;GPR55;TLR8;CDK5;CFTR;MCHR1;CHEK1;PSMB9;NFE2L2;DCUN1D1;MARK4;FFAR2;GRK5;P2RX4;KDM6B;HDAC9
PP12	Tellimagrandin I	MAOA;APEX1;TDP1;DUSP3;TRIM24;TTR;NTRK3;CTSD;CYP3A4;NR3C2;NR1A1;TLR4;F13A1;CDK5;GLRA1;IMPDH2;RPS6KA3;PDGFRA;EGLN1;STAT1;HSP90AA1;RORB;GPR35;NFKB1;GLS;SLC9A1;TDO2;CHRM3;GPR17;METAP2;KEAP1;NFE2L2;NOS2;ABCC1;MAP2K2;QRFPR;PIN1;PRSS1;PRCP;CFTR;TMEM173;MARK4;TFPI;PSMB9;CLK4;DNMT1;PLAT;ACACB;ACVR1B;ACACA;LYN;RPS6KA1;ACVRL1;STAT3;GPR6;CETP;ERAP1;ADRB1;P2RX7;RXFP1;HRH3;CCR1
PP13	Urolithin A	TDP1;APEX1;MAOA;GPR35;DUSP3;CTSD;RPS6KA3;TTR;TRIM24;NR1A1;CYP3A4;TLR4;GPR6;MAP2K2;CDK5;NOS2;NTRK3;METAP2;ACACA;NFE2L2;NR3C2;F13A1;GPR17;RORB;CLK4;BLM;NPC1;HSP90AA1;PLAT;PDGFRA;PIN1;BMP2K;CPT1B;SLC9A1;GUSB;ZAP70;ABCC1;TFPI;GLRA1;PRSS1;GRK5;NT5E;MIF;ADRB1;KEAP1;QRFPR;PRCP;ERN1;ERAP1;BRD2;LYN;DNMT1;IMPDH2;P2RX4;CFTR;NAMPT;CCR1;RPS6KA1;GPBAR1;GRB2;GLS
PP14	Granatin A	TDP1;APEX1;TRIM24;CTSD;NR1A1;MAOA;NTRK3;DUSP3;NR3C2;TMEM173;PTGS2;GLRA1;F13A1;NFKB1;EGLN1;CLK4;ABCC1;GPR17;RPS6KA1;TLR4;KEAP1;SLC9A1;BMP2K;METAP2;CDK5;CYP3A4;TTR;GPR55;SCD;TDO2;NFE2L2;PRCP;MAP2K2;HSP90AA1;QRFPR;LDHB;HSP90AB1;PDGFRA;TFPI;CETP;FCGRT;PIN1;P2RX4;TLR8;GLS;MARK4;PSMB9;ENPP1;GPBAR1;NT5E;ACACB;F2R;STAT3;ITK;ACACA;NNMT;CFTR;NR3C1;ZAP70;NOTUM;GRK5;AKR1C1;GRB2;DAO;FPRL2;PTK2B;TMPRSS6;TYRO3;CTSS;RORB;PTSG1;GPR6;NOS2;ACVRL1;DHODH;MTOR;ACVR1B;ULK3;CXCR6;BRD2
PP15	Methyl gallate	APEX1;TDP1;NFKB1;TTR;DUSP3;CDK5;GLRA1;CTSD;PDGFRA;GUSB;NPC1;MAOA;CYP3A4;STAT1;NFE2L2;RORB;MDM4;EPHB2;ERN1;NR1A1;RPS6KA3;NTRK3;MAP2K2;CLK4;TRIM24;TLR4;NOX1;QRFPR;PRCP;GPR35;SLC1A3;PLAT;CCR1;CFTR;CCR2;RAB9A;GPBAR1;GLS;IRAK1;METAP2;F13A1;ULK3;KLK7;NLK;NR3C2;CASP6;AVPR1B;GPR6
PP16	Catechin	NFKB1;ALPL;APEX1;TDP1;MAOA;NR1A1;TRIM24;EGLN1;TTR;CLK4;CTSD;ABCC1;NR3C2;NTRK3;RPS6KA1;GUSB;PRCP;TMEM173;F13A1;GLRA1;GRM5;LGALS3;ZAP70;HDAC8;ROCK1;OPRM1;ACVRL1;DUSP3;SLC9A1;TLR8;GPR55;QRFPR;CHRM5;STAT1;MARK4;PROC;CDK5;TFPI;KEAP1;FCGRT;GPBAR1;RORB;RPS6KA6;HDAC5;GPR17;ERAP1;CYP3A4;GLS;ITK;CASP8;METAP2;SCD;ACVR1B;IL23R;CHEK1;ACACA;ACE;KDM6B;DCUN1D1;FFAR2;BLM;DAT1;MAP2K2;NOS2;PSMB9;CETP;ADRB1;FPRL2
PP17	Casuarinin	TDP1;APEX1;MAOA;DUSP3;CTSD;TTR;TRIM24;NR1A1;NTRK3;NR3C2;DNMT1;TFPI;F13A1;CYP3A4;GLRA1;ABCC1;PIN1;CDK5;PDGFRA;GPR17;TLR4;LYN;CHRM3;CHRM2;STAT1;CFTR;NFKB1;ADRB1;EGLN1;IMPDH2;RORB;HDAC5;NFE2L2;QRFPR;ACVRL1;PRCP;STAT3;AXL;METAP2;BLM;PLAT;NOTUM;SLC9A1;MIF;CETP;MAP2K2;BRD2;ACACB;HDAC2;HRH3;PRSS1;GRK5;LDHB;CHRM1;HSP90AB1;P2RX7;GPBAR1;ACVR1B;ACACA;RPS6KA1;NPC1;CYSLTR2;GUSB;GPR35;TDO2;HSP90AA1;GLS
PP18	Corilagin	APEX1;L3MBTL1;POLB;RAD52;TDP1;MAOA;DUSP3;CTSD;TRIM24;TTR;NTRK3;NR1A1;RPS6KA3;NR3C2;TLR4;CYP3A4;HSP90AA1;PDGFRA;F13A1;GLRA1;GPR17;SLC9A1;CDK5;NFKB1;GPR35;CFTR;IMPDH2;RORB;PIN1;TFPI;STAT1;GPR6;PRCP;METAP2;ABCC1;MAP2K2;TMEM173;QRFPR;CHRM3;DNMT1;PRSS1;ACACA;NFE2L2;ACACB;ADRB1;KEAP1;CBX4;MARK4;PLAT;PTGS2;HDAC5;ACVRL1;PSMB9;LDHB;BRD2;RPS6KA1;MIF;GLS;GRK5;ACVR1B;HSP90AB1;LYN;GPBAR1;EGLN1;TDO2
PP19	Castalin	TDP1;APEX1;CTSD;TRIM24;DUSP3;NR1A1;BLM;F13A1;MAOA;HSP90AA1;NFKB1;NR3C2;GLRA1;TTR;RPS6KA1;NTRK3;CDK5;GRK5;MAP2K2;GPR35;DNMT1;PIN1;RPS6KA3;ABCC1;DHODH;TFPI;NFE2L2;SCD;ZAP70;ACACA;PDGFRA;ENPP1;MIF;VDR;TDO2;PLAT;PSMB9;MINK1;QRFPR;PRCP;METAP2;TLR4

		;GPR17;BMP2K;P2RX4;ITK;FCGRT;EGLN1;STAT3;DAO;CFTR;GUSB;GPCR40;TYRO3;ERAP1;RORB;ADRB1;NPC1;ACACB
PP20	(-)-Gallocatechin gallate	BCL2;RAD52;TDP1;BCL2;TTR;POLB;PGAM1;DNMT1;STAT1;BACE1;APEX1;TDP1;FUT7;MAOA;NFKB1;BLM;ABCC1;CHRM3;CTSD;RPS6KA3;TRIM24;DUSP3;CYP3A4;NTRK3;NR3C2;SLC9A1;NR1A1;QRFPR;OPRM1;PDGFRA;EGLN1;GLRA1;TFPI;GUSB;F13A1;RPS6KA1;CFTR;TLR4;CDK5;HDAC5;ROCK1;RORB;NOS2;MARK4;LYN;METAP2;CHRM2;ACVR1B;PRCP;GPR17;ACACA;CHRM1;PIN1;PTSG1;ACVRL1;GPBAR1;MAP2K2;ERAP1;CCR1;RPS6KA6;KEAP1;NFE2L2;GLS;LGALS3;IL23R;IMPDH2;CBX4;PLAT;AXL
PP21	Granatin B	TDP1;APEX1;MAOA;DUSP3;CTSD;TRIM24;NTRK3;CYP3A4;TFPI;TLR4;CDK5;NR1A1;PIN1;NR3C2;TTR;PDGFRA;METAP2;NFKB1;CFTR;F13A1;SLC9A1;GPR6;MAP2K2;QRFPR;PRCP;STAT1;ABCC1;GPR17;GUSB;DNMT1;KEAP1;GPBAR1;STAT3;LDHB;CHRM2;IMPDH2;ACACB;RPS6KA1;MARK4;CETP;RPS6KA3;PRSS1;MIF;HDAC5;ACACA;CLK4;NFE2L2;CHRM1;TYRO3;NR3C1;RORB;GLRA1;ACVR1B;TMPRSS6;TMEM173;HSP90AA1;EGLN1;BRDT;CBX4;TRPM8;FPRL2;CHRM3;NOTUM;TRPA1;PLAT;GLS;DRD3;PTGS2;AXL;ADRB1;SCD;NNMT;HDAC8;PSMB9;FCGRT;ULK3;CCR1;BRD2;ERAP1
PP22	Valoneic acid dilactone	APEX1;TDP1;DUSP3;NR1A1;TTR;TRIM24;GPR35;RORB;MAOA;AKR1C1;ERN1;NR4A1;CLK4;CTSD;RAB9A;MDM4;NPC1;ADRB1;GLRA1;AKR1C2;MIF;LGALS3;PTGS2;CYP3A4;HSP90AA1;CPT1B;F13A1;PIN1;CETP;CCR1;PRCP;P2RX4;MAP2K2;ACACA;RPS6KA3;ERAP1;ZAP70;NTRK3;NFE2L2;ACACB;DHODH;CDK5;GPR6;GLS;PLAT;CASP6;GPBAR1;STAT1;CHRM5;PREP;QRFPR;FLT1;RPS6KA1;GUSB;CHRM3;MC5R;MC3R;CHKA;PDGFRA;AXL;RXFP1;KCNK9
PP23	Ellagic acid	APEX1;TDP1;POLH;POLI;GPR35;RECQL;SMAD3;POLB;AKR1B1;INSR;GALNT2;NUAK1;MET;ELAVL3;KDR;DUSP3;TRIM24;MAOA;ERN1;TTR;NR1A1;CLK4;CTSD;GLRA1;F13A1;NR4A1;GPBAR1;AKR1C1;LGALS3;PRCP;BMP2K;ACACA;NTRK3;RPS6KA3;MAP2K2;NPC1;RPS6KA1;MIF;NR3C2;METAP2;EGLN1;CDK5;NOS2;GUSB;P2RX4;DAO;GLS;QRFPR;HDAC8;RORB;CFTR;TYRO3;ADRB1;CYP3A4;NFE2L2;ZAP70;HSP90AA1;TBXA2R;FCGRT;ACACB;PIN1;MDM4;PLAU;ERAP1;TFPI;SCD;GRK5;GPR17;CLK2;AXLGPR6
PP24	kaempferol-3-O-β-D-glucopyranoside	ACHE;TDP1;APEX1;BLM;CTSD;RPS6KA3;DUSP3;TTR;NR1A1;CLK4;NFKB1;TRIM24;ESR2;MAOA;GLRA1;F13A1;GRK5;HSP90AA1;GPR17;NTRK3;LDHB;RORB;NR3C2;GPBAR1;CHRM1;NFE2L2;QRFPR;PIN1;HDAC2;CFTR;TFPI;CXCR4;METAP2;TLR4;MTOR;FCGRT;HDAC5;NR4A1;NOS2;SLC1A2;AVPR1B;SCD;ABCC1;PRCP;KDM4C;PLAT;TYRO3;GPR6;STAT1;CHRM3;CBX4;SLC1A1;ZAP70;GUSB;ACACA;ADRB1;TBXA2R;RXFP1;CHEK1;PLAU;MIF;DCUN1D1;P2RX4;ERAP1;AKR1C1
PP25	3-Glucosylquercetin	SMN1;TDP1;APEX1;CTSD;NR1A1;MAOA;DUSP3;RPS6KA3;TTR;CLK4;NFKB1;TRIM24;BLM;MIF;GLRA1;LDHB;GPR17;GRK5;F13A1;GPBAR1;NFE2L2;NR3C2;HSP90AA1;ACHE;RORB;NTRK3;CFTR;GUSB;METAP2;ADRB1;MTOR;TFPI;ABCC1;CHRM1;RXFP1;TLR4;NOS2;SCD;HDAC2;NR4A1;PLAT;PIN1;GPR6;PRCP;STAT1;QRFPR;TYRO3;ACACA;CHRM3;CBX4;HDAC5;P2RX4;CYP3A4;SLC1A2;DCUN1D1;AVPR1B
PP26	5-hydroxymethylfurfural	TRIM24;APEX1;HDAC5;CTSD;ERN1;CLK4;NFE2L2;TTR;NFKB1;RORB;DUSP3;STAT1;HDAC8;TDP1;NPC1;GRK5;ABCC1;NR3C2;PRCP;RAB9A;GPBAR1;GLRA1;F13A1;GPR35;NTRK3;VDR;PIN1;BMP2K;HDAC3;SCD;AXL;PRSS1;METAP2;SLC40A1;TBXA2R;FLT1;QRFPR;HDAC11;HDAC7;LGALS3;AHCYL2;CBX4;CYSLTR2
PP27	Pelargonidin	APEX1;NFKB1;CTSD;BLM;NTRK3;TDP1;SLC40A1;CLK4;GPR55;NR3C2;TRIM24;PTSG1;NFE2L2;GPR17;PSMB9;CYSLTR2;RXFP1;CHRM5;DCUN1D1;KEAP1;ITK;ABCC1;GLRA1;AHCYL2;CYP3A4;PRSS1;SCD;RORB;TLR4;ADORA2B;FPRL2;PIN1;NT5E;HDAC10;DUSP3;MAP3K14;CHRM4;CHRM2;MTOR;HSP90AB1;ERAP2;ANPEP;MAOA;TFPI;CASP8;TMEM173;TMPRSS6;PDGFRA;NOTUM;HPRT1;P2RX7;STAT1;GRK5;CHKA;NR4A1;SLC1A2;DYR;SLC9A1;EPHX1;NAMPT;CCR1;HDAC7

PP28	3,3'-Di-O-methylellagic acid 4'-glucoside	APEX1;TDP1;BLM;DUSP3;TRIM24;CTSD;CLK4;HSP90AA1;NFKB1;NFE2L2;NTRK3;GPR35;NR1A1;MAOA;TTR;MIF;NR3C2;TLR4;GLRA1;F13A1;SCD;RXFP1;NOS2;ZAP70;GPBAR1;NR4A1;PRCP;ADRB1;PLAU;HDAC2;CYP3A4;ENPP1;DNMT1;RORB;MDM4;METAP2;TYRO3;KEAP1;ANPEP;ACACB;GPR6;CCR1;GRK5;GLS;P2RX7;ACVRL1;CCR2;LDHB;ALK;HSP90AB1;PLAT;TFPI;ITK;GPR55
PP29	Apigenin	APEX1;NFKB1;CTSD;TRIM24;HDAC2;NR3C2;NFE2L2;HSP90AB1;BLM;CLK4;NTRK3;ACHE;MAOA;HSP90AA1;TLR4;GPR55;PDGFRA;HDAC10;GLRA1;DUSP3;MAP2K2;GPR35;SCD;ACACA;CFTR;RORB;RXFP1;ACACB;PLAU;HDAC5;ITK;PRCP;DCUN1D1;TYRO3;GPR6;SLC9A1;QRFPR;NR4A1;TFPI;METAP2;CYSLTR2;WDR5;CETP;F13A1;TMPRSS6;TMEM173;GRK5;ABCC1;CYP3A4;PTSG1;HPRT1;RPS6KA1;GPR17;GPBAR1;SLC40A1;CTSB;FPRL2;NR1A1;CCR1;PROC;NOTUM;KAT6A;HDAC11;MTOR
PP30	5-hydroxymethylfuran-3-carboxylic acid	RAB9A;NPC1;HDAC5;TRIM24;TTR;CTSD;APEX1;RORB;NFE2L2;NFKB1;STAT1;GLRA1;HDAC7;MDM4;BLM;NR4A1;ATG4B;PSMB9;GPR35;CYP3A4;CDK5;ERAP1;TBXAS1;PRSS1;ACACA;SLC40A1;ABCC1;PIN1;F13A1;CASP6;APLNR;NOX1;LGALS3;PDGFRA;FLT1;METAP2;DUSP3;KDM4A;AXL;CLK4;CFTR;CPT1B;KCNK9;SLC9A1;TYRO3;PRCP;MAP2K2;NR3C2;AKR1C2;HDAC8
PP31	Cyanidin	APEX1;NFKB1;TRIM24;CLK4;DUSP3;NTRK3;ACVRL1;HSP90AB1;CTSD;ALK;GLRA1;NR3C2;CLK2;NR1A1;NFE2L2;KEAP1;ADRB1;EGLN1;PRCP;CCR1;ZAP70;CYP3A4;TFPI;CCR2;RORB;SCD;ACACB;SLC9A1;F13A1;AKR1C1;GLS;BLM;TLR4;GPR6;HDAC8;CYSLTR2;NOX1;ALOX5;MIF;CFD;AXL;RXFP1;QRFPR;CFTR;PTK2B;TTR;METAP2;ABCC1;KDM4A;ENPP1;F7;NOTUM;NAMPT;ITK;CETP;GPBAR1
PP32	Pelletierine	TDP1;NFKB1;CTSD;CLK4;BLM;ACACA;TLR4;CYP3A4;SLC5A1;APEX1;NR3C2;METAP2;PDE7A;NTRK3;TMPRSS6;ADRB1;PLAT;GPR55;WDR5;GLRA1;HDAC10;TERT;SPHK1;CCR1;ADORA2B;HSP90AA1;FPRL2;MTOR;SLC40A1;SLC9A1;CTSK;CFTR;CTSB;ZAP70;BMP2K;GPR35;DAT1;P2RX7;HDAC11;TRIM24;STAT3;EPHX1;RORB;NT5E;CLK2;CCR2;NFE2L2;PDGFRA;PARP2;SCD;QRFPR;FCGRT;AHCYL2;HCAR2;PSMB9;PNR;PLG;JAK3;MMP7;GPR17;KCNK9;MC5R;MC4R;VCP;CYSLTR2;5HTT;PTK2B
PP33	Isopelletierine	TDP1;NFKB1;CTSD;CLK4;BLM;ACACA;TLR4;CYP3A4;SLC5A1;APEX1;NR3C2;METAP2;PDE7A;NTRK3;TMPRSS6;ADRB1;PLAT;GPR55;WDR5;GLRA1;HDAC10;TERT;SPHK1;CCR1;ADORA2B;HSP90AA1;FPRL2;MTOR;SLC40A1;SLC9A1;CTSK;CFTR;CTSB;ZAP70;BMP2K;GPR35;DAT1;P2RX7;HDAC11;TRIM24;STAT3;EPHX1;RORB;NT5E;CLK2;CCR2;NFE2L2;PDGFRA;PARP2;SCD;QRFPR;FCGRT;AHCYL2;HCAR2;PSMB9;PNR;PLG;JAK3;MMP7;GPR17;KCNK9;MC5R;MC4R;VCP;CYSLTR2;5HTT;PTK2B
PP34	pseudopelletierine	CTSD;TRIM24;NFKB1;GPR55;STAT3;ACACA;NTRK3;GLRA1;TLR4;BLM;HSP90AB1;DAT1;TDO2;MAOA;TMPRSS6;NR3C2;PDE7A;HDAC10;SLC9A1;CYSLTR2;DCUN1D1;HRH3;TLR8;LDHA;CTSB;LDHB;CYP3A4;ABCC1;EGLN1;ACHE;IRAK4;P2RX7;CFTR;MTOR;GPBAR1;EZH2;TYRO3;ACACB;BMP2K;WDR5;CLK4;ALK;NFE2L2;PRCP;DRD3;AXL;RORC;SCD;PTK2B;RPS6KA1;TDP2;HDAC8;FPRL2;KLK7;NR4A1;ATG4B;DUSP3;SPHK1;MMP9;PDGFRA;CCR5;CCR1;ULK1;PHF8;MC5R;FKBP5;TERT;PROC;PLAT;TRPA1;5HTT;CARM1;ITK;ERAP2;LGALS3;MC3R
PP35	Oleanic Acid	AKR1B10;BLM;GPR55;PTSG1;NFKB1;APEX1;CLK4;TRIM24;HSP90AB1;NR3C2;CTSD;NTRK3;STAT3;TLR4;CYP3A4;GLRA1;ITK;TLR8;SLC9A1;NR4A1;PDGFRA;CHRM4;CYSLTR2;TERT;MARK4;CDK5;FPRL2;HDAC7;CHRM1;HSP90AA1;RPS6KA1;PRCP;PROC;F11;PTK2B;TMEM173;WDR5;CHRM5;MTOR;ATG4B;ACACB;CHRM2;PLAT;DUSP3;PSMB9;F13A1;HDAC11;TFPI;DCUN1D1;SLC40A1;ULK3
PP36	kaempferol	CYP1B1;CISD1;HSD17B2;APEX1;TDP1;RPS6KA3;CTSD;TTR;MAOA;ESR2;DUSP3;NR1A1;TRIM24;CLK4;GLRA1;RPS6KA1;GPBAR1;TBK1;F13A1;RPS6KA6;ERN1;STAT1;HDAC8;AKR1C1;PIN1;ACVR1B;CXCR4;FCGRT;CDK5;LGALS3;NR4A1;RORB;BMP2K;GRK5;PRCP;LDHB;NTRK3;AVPR1B;HDAC2;METAP2;MELK;CFTR;EGLN1;GSK3B;MAPKAPK2;ACACA;NR3C2;QRFPR;ERAP1;NPC1;NLK;AKR1C2;MTOR;P2RX4;TBXA2R;GUSB;GPR17;CHKA;MAP2K2;ZAP70;

		NOS2;CBX4;MAP3K11;MINK1;GLS;KDM4A;TFPI;NFKB1;MC5R;MIF;ULK3;SLC1A2;DCUN1D1;SLC1A1;IMPDH2;SCD;MME;TYRO3;PLAT;CHRM5;ADRB1;AKR1B1
PP37	β-Sitosterol	TDP1;NFKB1;CTSD;CLK4;PTSG1;NR3C2;GPR55;GLRA1;APEX1;NTRK3;HDAC8;ITK;ESR2;STAT3;TRIM24;MTOR;VDR;TMPRSS6;ABCC1;ACACA;DAT1;ANPEP;DYR;PROC;DCUN1D1;FPRL2;PRCP;CFTR;ADORA2B;NFE2L2;HDAC2;CLK2;CCR1;CYSLTR2;METAP2;SLC9A1;TDO2;CHRM4;RPS6KA1;SCD;CTSB;ACACB;CHRM5;HDAC10;CTSK;CDK7;SPHK1;EZH2;P2RX7;LDHA;ERAP1;TLR4;RXFP1;SLC40A1;PSMB9;BMP2K;PAK4;CBX4;QRFPR;CASP8
PP38	luteolin	TTR;CYP1B1;AKR1B1;TDP1;APEX1;MAOA;NR1A1;CTSD;GUSB;DUSP3;TRIM24;GLRA1;EGLN1;CLK4;GPBAR1;F13A1;MIF;METAP2;MAPKAPK2;ESR2;LGALS3;RPS6KA1;ERN1;STAT1;CDK5;NPC1;PIN1;HDAC8;PRCP;RORB;NR4A1;TBK1;CDK1;CFTR;P2RX4;RPS6KA3;GPR17;GRK5;LDHB;CYP3A4;FCGRT;ANPEP;CHKA;NTRK3;GSK3A;ACACA;RPS6KA6;NR3C2;CXCR4;CBX4;GLS;AVPR1B;ACVR1;MTOR;EIF2AK1;ADRB1;HSP90AA1;HDAC2;ACVR1B;MME;ERAP1;F2R;TFPI;ZAP70;DCUN1D1;QRFPR;MC5R;TYRO3;KDM4C
PP39	quercetin	AKR1B1;CYP1B1;ABCG2;NPSR1;POLB;KDR;MET;RECQL;IP6K2;ALOX5;ALOX15;AXL;TDP1;APEX1;MAOA;TTR;NR1A1;CTSD;RPS6KA3;DUSP3;TRIM24;CLK4;ERN1;GLRA1;GPBAR1;TBK1;RPS6KA1;MIF;F13A1;STAT1;GSK3B;LDHB;CDK5;LGALS3;RPS6KA6;EGLN1;GUSB;NPC1;RORB;BMP2K;NR4A1;ACVR1B;PRCP;GRK5;CFTR;METAP2;HDAC8;ACACA;NTRK3;PIN1;NR3C2;ESR2;MAPKAPK2;AKR1C1;ADRB1;P2RX4;GPR17;ERAP1;MELK;AVPR1B;HDAC2;CHKA;FCGRT;MTOR;NFKB1;CLK2;NOS2;MAP3K11;TBXA2R;MAP2K2;ULK3;CYP3A4;DCUN1D1;CBX4;ANPEP;MC5R;CPT1B;SCD;TFPI;PLAT;MME;GLS;CXCR4;NFE2L2;NLK;AKR1C2

Table S4. The differentially expressed genes in GSE36701.

ID	logFC	AveExpr	t	P.Value	adj.P.Val	B
CAPN8	0.91740	6.97675	5.04645	0.00000	0.00344	4.42531
LOC101928820	0.91902	7.99150	4.58462	0.00002	0.00979	2.81646
RP11-568N6.1	0.61478	6.20790	4.51791	0.00002	0.01163	2.59098
RP11-676J12.4	0.50878	6.28413	4.44819	0.00003	0.01266	2.35731
RP5-1027O15.1	-0.51903	2.48300	-4.27262	0.00005	0.01813	1.77838
UPP1	0.58869	5.53379	3.98208	0.00015	0.02966	0.85194
CIART	-0.62097	3.11123	-3.84741	0.00024	0.03711	0.43683
FAM187B	-0.56471	2.33348	-3.83105	0.00025	0.03881	0.38704
MAB21L3	0.55046	4.27057	3.82880	0.00025	0.03881	0.38021
LOC101929988	0.53821	5.31517	3.79522	0.00028	0.04085	0.27852
POPDC2	0.53546	2.43313	3.67700	0.00043	0.04975	-0.07875
PIWIL1	1.03022	3.60599	3.64793	0.00047	0.05059	-0.16029
SERTAD3	-0.56086	5.26353	-3.63145	0.00049	0.05063	-0.20863
LOC101928068	0.56480	3.59887	3.61745	0.00052	0.05063	-0.24958
SERPINB5	1.08620	5.60731	3.57747	0.00059	0.05465	-0.36590
PER3	-0.51481	5.38338	-3.55867	0.00063	0.05693	-0.42031
CCL13	0.55051	4.41968	3.54808	0.00065	0.05797	-0.45085
LOC100288152	0.74515	5.76724	3.53831	0.00067	0.05937	-0.47898
PRKXP1	0.62288	4.67751	3.50705	0.00074	0.06247	-0.56859
SLC28A2	-0.69166	4.53942	-3.44531	0.00091	0.06672	-0.74389
USP2	-0.51738	4.03304	-3.38083	0.00111	0.07321	-0.92454

CD177	-0.98603	7.25588	-3.34434	0.00125	0.07601	-1.02567
CYP2C18	0.53588	3.94593	3.32123	0.00134	0.07912	-1.08931
OR2B6	-0.56426	1.78828	-3.31570	0.00141	0.08031	-1.11516
RP11-210M15.2	-0.79479	1.08770	-3.30830	0.00147	0.08135	-1.14215
SLC16A9	0.50426	8.38248	3.26290	0.00161	0.08522	-1.24842
RP11-90C4.2	0.63725	2.37935	3.23874	0.00175	0.08742	-1.31471
AF090939	0.61121	2.76090	3.19560	0.00199	0.09118	-1.42941
LINC00277	-1.07731	1.95289	-3.18418	0.00207	0.09252	-1.46047
FAM19A1	-0.65729	3.25177	-3.16570	0.00218	0.09471	-1.50887
USP27X-AS1	-1.39892	1.53151	-3.16011	0.00228	0.09697	-1.52948
LOC100505811	-0.50461	2.08961	-3.14088	0.00235	0.09770	-1.57440
ALOX12B	1.11896	1.10513	3.14237	0.00258	0.10183	-1.60038
MGMT	-0.50626	4.16990	-3.12021	0.00250	0.10101	-1.62870
CLC	-0.81883	4.81813	-3.11014	0.00258	0.10183	-1.65503
DCSTAMP	0.52651	2.80620	3.07144	0.00291	0.10678	-1.75577
CAPZA3	-0.82475	0.95058	-3.06812	0.00303	0.10906	-1.76715
IQCF6	0.85233	1.39681	3.05754	0.00304	0.10906	-1.79139
TFAP2A	0.54238	3.54661	3.01535	0.00342	0.11586	-1.89976
SERPINB7	0.79216	1.95330	2.96558	0.00400	0.12565	-2.02462
TMEM235	1.08618	1.65379	2.94914	0.00448	0.13031	-2.06878
MRGPRX3	-0.62260	3.39355	-2.94764	0.00418	0.12792	-2.07093
USP26	0.61377	2.33654	2.93733	0.00431	0.12896	-2.09671
NMBR	-0.79749	1.14858	-2.92865	0.00471	0.13308	-2.11632
GRID1-AS1	0.63705	2.10943	2.90912	0.00469	0.13308	-2.16620
LOC100996760	0.62224	4.62401	2.89623	0.00485	0.13511	-2.19881
LOC100129112	-0.57297	1.62417	-2.85508	0.00557	0.13996	-2.29362
SLC4A3	-0.78016	1.90014	-2.83202	0.00588	0.14399	-2.35277
LOC100996412	0.52658	3.67989	2.82240	0.00599	0.14527	-2.37934
CARD18	-1.11049	1.21460	-2.81242	0.00633	0.14914	-2.39347
BC021061	-0.68278	2.27836	-2.80396	0.00633	0.14914	-2.42261
SCNN1G	-0.53064	3.86213	-2.80140	0.00636	0.14927	-2.42999
RP11-1081M5.2	-1.02120	1.00802	-2.77659	0.00708	0.15604	-2.47429
SIX6	-0.53378	2.60733	-2.76028	0.00715	0.15694	-2.52687
RP11-506N2.1	-0.54558	2.22686	-2.75224	0.00733	0.15941	-2.54441
LINC00029	0.58946	2.11990	2.74218	0.00756	0.16115	-2.56655
OR10J1	-0.77723	0.99791	-2.73466	0.00809	0.16497	-2.56717
LOC101929662	-0.93393	0.95026	-2.73471	0.00795	0.16404	-2.57153
APOA5	0.85193	0.95289	2.72337	0.00828	0.16606	-2.59349
LINC01365	-0.79324	2.02620	-2.71252	0.00832	0.16634	-2.62822
RP11-307P5.2	-0.50649	2.72848	-2.69910	0.00849	0.16860	-2.66892
PNLIPRP2	0.85956	7.14801	2.69772	0.00849	0.16860	-2.67558
HIST1H4G	-0.93876	1.51040	-2.68759	0.00893	0.17212	-2.68418
SYT2	-0.74605	1.35662	-2.68438	0.00906	0.17288	-2.68879
RDH8	-0.52679	1.61368	-2.68127	0.00899	0.17249	-2.70450
LOC102725116	0.58097	1.49780	2.66506	0.00937	0.17563	-2.74403
PRAME	-0.88581	1.45742	-2.65069	0.00981	0.17869	-2.77086

IL23A	0.55561	2.61571	2.65380	0.00959	0.17817	-2.77526
TEKT3	-0.58630	2.32805	-2.64797	0.00975	0.17857	-2.78880
DUOX2	0.76736	6.99136	2.64475	0.00981	0.17869	-2.79809
RDH12	0.91327	0.95093	2.62242	0.01131	0.18850	-2.80506
LOC100131508	0.60925	1.80508	2.63256	0.01014	0.18176	-2.82600
LINC01169	0.98097	0.95593	2.61433	0.01124	0.18788	-2.83102
HBB	-0.78195	6.47193	-2.62557	0.01033	0.18291	-2.84197
ECHDC3	0.95367	2.04800	2.61407	0.01080	0.18503	-2.85566
BATF3	0.55859	2.34353	2.61079	0.01075	0.18446	-2.87559
LOC101928173	0.51255	1.89997	2.60210	0.01105	0.18700	-2.89105
OR2W1	0.61211	1.94626	2.59525	0.01121	0.18777	-2.91076
SPHKAP	-0.70368	2.29488	-2.58254	0.01172	0.19201	-2.92863
C15orf45	0.78033	0.83165	2.55633	0.01277	0.19835	-2.97183
MST1	-0.69781	4.25517	-2.55316	0.01254	0.19740	-3.00517
LOC283038	0.56831	2.10879	2.54970	0.01271	0.19814	-3.00824
CRNDE	0.56371	4.74361	2.54864	0.01270	0.19814	-3.01522
RP11-400N9.1	0.53745	2.29690	2.53492	0.01319	0.20102	-3.04332
FAM170A	0.55629	3.07010	2.52444	0.01356	0.20316	-3.06628
RAB38	0.61341	3.35338	2.52382	0.01356	0.20316	-3.07019
C18orf12	-0.51470	3.91187	-2.51874	0.01374	0.20413	-3.08139
LRRC71	-0.55623	1.88786	-2.49614	0.01471	0.21155	-3.11776
BANF2	-0.50449	2.81352	-2.49487	0.01468	0.21155	-3.12869
NPAS4	-0.67396	0.50854	-2.47229	0.01596	0.21995	-3.14711
DKK1	0.55188	2.99157	2.48441	0.01503	0.21304	-3.15656
SBSN	0.74395	2.10105	2.47831	0.01532	0.21396	-3.16466
SNRPN	-0.54442	2.34517	-2.44433	0.01670	0.22479	-3.24054
CTD-2611O12.6	-0.63712	1.74645	-2.43103	0.01731	0.22901	-3.26618
DQ580846	0.53555	1.88353	2.42547	0.01756	0.23061	-3.27801
ABCD2	0.50592	2.44341	2.40568	0.01844	0.23504	-3.32280
CUBN	-0.59767	2.85986	-2.39778	0.01879	0.23583	-3.34231
MPZ	-0.54526	3.80267	-2.39046	0.01917	0.23786	-3.35471
MST1L	-0.72060	7.71811	-2.38340	0.01949	0.23864	-3.37260
SLC38A5	-0.88123	1.69084	-2.37492	0.02003	0.24221	-3.37862
PKD1P1	0.70236	1.75579	2.37425	0.01994	0.24166	-3.39178
LOC100505776	-0.51244	2.07619	-2.36834	0.02034	0.24382	-3.39512
FOXN3-AS2	-0.79800	0.64081	-2.32463	0.02386	0.25835	-3.41043
LHX8	-0.60868	1.61590	-2.36204	0.02063	0.24489	-3.41133
LOC101929550	0.51272	2.12681	2.35829	0.02079	0.24565	-3.42215
HLA-DQB1	-0.51398	4.86587	-2.34162	0.02165	0.25062	-3.45971
LOC100507616	0.67578	3.00505	2.33482	0.02205	0.25150	-3.47053
AY927499	-0.90678	0.99507	-2.30614	0.02434	0.26006	-3.47780
DCST1	-1.25404	-0.07359	-2.27264	0.02764	0.27487	-3.49056
SCT	-1.10073	0.56653	-2.28931	0.02570	0.26719	-3.49282
RXRG	-0.51010	1.37627	-2.29265	0.02466	0.26195	-3.54038
CTC-471C19.1	0.57632	1.82396	2.29509	0.02434	0.26006	-3.55180

CXorf31	-1.44065	0.14380	-2.21834	0.03288	0.29213	-3.56465
U47924.29	-0.53276	2.37828	-2.28461	0.02504	0.26394	-3.56675
LINC00403	-0.55000	2.30835	-2.28243	0.02511	0.26421	-3.57762
LOC728196	-1.02532	0.97910	-2.22980	0.02963	0.28170	-3.60670
LOC102725408	-0.85790	0.47238	-2.22670	0.02991	0.28222	-3.60936
LOC101927133	0.65655	1.60113	2.25007	0.02733	0.27440	-3.62920
MMP7	0.53778	1.71069	2.25028	0.02720	0.27440	-3.63840
KCNAB3	0.61824	1.83527	2.24067	0.02796	0.27530	-3.64780
LINC00606	0.61412	1.20364	2.20003	0.03124	0.28667	-3.69374
CSRP3	0.88064	1.54293	2.16246	0.03537	0.30055	-3.69988
REEP6	-0.76228	0.96480	-2.19299	0.03182	0.28844	-3.70517
CFHR4	-0.57268	1.53371	-2.20338	0.03071	0.28428	-3.71029
LINC00960	0.58207	3.82523	2.21540	0.02953	0.28146	-3.71471
GALNT14	0.59496	1.52893	2.21232	0.02979	0.28170	-3.71737
HPDL	0.53021	2.90677	2.20782	0.03011	0.28234	-3.72622
ZNF876P	0.58948	1.94790	2.20747	0.03014	0.28234	-3.72671
NPPC	0.62478	2.40640	2.19764	0.03097	0.28598	-3.73558
DOC2A	-0.60757	1.37474	-2.16437	0.03374	0.29520	-3.77951
CPA4	0.51022	2.64688	2.17767	0.03233	0.28999	-3.78853
CD1A	0.59435	3.37991	2.17265	0.03272	0.29168	-3.79826
A4GNT	1.02251	0.12162	2.08582	0.04322	0.32698	-3.80262
LOC101929926	0.58209	2.00442	2.14304	0.03518	0.30012	-3.84801
SAA3P	-0.50881	2.06609	-2.13768	0.03558	0.30071	-3.86174
LINC01282	-0.91543	0.70087	-2.09247	0.04041	0.31845	-3.88053
TCTE1	0.52181	1.89287	2.11890	0.03727	0.30651	-3.88997
C2orf83	-0.68830	1.14871	-2.11751	0.03739	0.30681	-3.89281
LOC100130331	-0.54449	1.46183	-2.11291	0.03780	0.30896	-3.90148
TPRXL	0.53123	1.62032	2.10838	0.03816	0.31050	-3.91370
RP11-102M11.2	-0.99212	1.18177	-2.06038	0.04384	0.32903	-3.91744
CIB4	-0.82346	0.61695	-2.04192	0.04562	0.33439	-3.94753
NFKBIL1	-0.54711	1.66112	-2.08557	0.04023	0.31778	-3.95643
IL27	0.65457	1.76784	2.07498	0.04123	0.32045	-3.97611
GPX5	-0.59863	0.92866	-2.06327	0.04249	0.32489	-3.98590
LOC340074	0.63147	1.58150	2.04230	0.04472	0.33199	-4.01185
AX746710	-0.60704	1.92317	-2.04074	0.04474	0.33199	-4.02710
PAX8-AS1	0.59488	5.78696	2.04320	0.04427	0.33075	-4.04247
TPRG1	0.82186	1.63970	2.01836	0.04732	0.33961	-4.04722
MSANTD1	-0.56622	2.32422	-2.01490	0.04744	0.33961	-4.07408
LEMD1	0.60271	1.41550	2.00474	0.04849	0.34205	-4.09590
AADACL2	0.63324	2.74122	1.99550	0.04934	0.34474	-4.12908

Table S5. The differentially expressed genes in GSE14841.

ID	logFC	AveExpr	t	P.Value	adj.P.Val	B
RP11-692P14.1	-0.62826	3.56302	-7.62012	0.00001	0.06555	3.42538
DISP2	1.07499	6.17535	7.57115	0.00001	0.06555	3.37827

UBE2S	-0.53550	6.30090	-7.52382	0.00001	0.06555	3.33242
YWHAE	-1.09396	8.27688	-7.40945	0.00001	0.06555	3.22025
ND6	1.13486	7.15125	7.28706	0.00002	0.06555	3.09806
PROC	0.52629	4.03737	6.97466	0.00002	0.07656	2.77581
MIR10A	-0.53279	4.72769	-6.76314	0.00003	0.07656	2.54897
REPIN1	-0.79641	6.35952	-6.68773	0.00003	0.07656	2.46635
HNRNPL	0.96734	7.61206	6.65782	0.00003	0.07656	2.43333
SNRNP40	0.79151	6.01282	6.64263	0.00004	0.07656	2.41651
GLP2R	-0.52817	3.82867	-6.45537	0.00005	0.08995	2.20597
SMIM2-AS1	0.56685	4.88654	6.07278	0.00008	0.09549	1.75759
CYP26B1	-0.60578	4.81961	-6.05585	0.00008	0.09549	1.73718
LOC253044	-0.51729	3.33276	-6.04886	0.00008	0.09549	1.72874
NDRG2	-0.60665	6.12684	-6.02615	0.00008	0.09549	1.70125
EFNA1	-0.68481	8.59795	-6.01941	0.00008	0.09549	1.69307
SLC9A3	-0.88116	4.76414	-5.98988	0.00009	0.09549	1.65716
PPP1R36	0.65465	7.08242	5.87569	0.00010	0.09780	1.51687
SULT2A1	-0.88674	9.35639	-5.82692	0.00011	0.09780	1.45627
BSG	-1.00420	8.38356	-5.82025	0.00011	0.09780	1.44795
TMEM259	-0.56155	5.04325	-5.71106	0.00013	0.10510	1.31063
RAB5C	-0.83180	8.33004	-5.56077	0.00017	0.10959	1.11822
LOC340184	-0.80351	4.43757	-5.55151	0.00017	0.10959	1.10623
GGT6	0.68840	7.10219	5.53002	0.00017	0.10959	1.07837
PIP5K1A	-0.57530	6.11227	-5.47258	0.00019	0.10959	1.00348
TIGD3	0.57149	5.13416	5.40738	0.00021	0.10959	0.91776
TRIM25	-0.51766	7.70925	-5.39962	0.00021	0.10959	0.90751
RP11-96D1.11	0.53831	6.15024	5.27606	0.00026	0.11847	0.74287
TRIM3	-0.51661	5.26485	-5.22013	0.00028	0.11847	0.66746
NGEF	-0.53630	7.12173	-5.17359	0.00030	0.11847	0.60429
DSERG1	-0.55856	6.00152	-5.17221	0.00030	0.11847	0.60242
LOC100505915	-0.90808	5.66308	-5.13627	0.00032	0.11847	0.55338
EP300-AS1	0.53273	3.27088	5.09513	0.00034	0.11847	0.49696
SRPR	-0.61261	7.64230	-5.03530	0.00037	0.11847	0.41439
TRG-AS1	-0.56916	6.71627	-4.98117	0.00041	0.12152	0.33915
HBP1	-0.59740	5.38134	-4.92099	0.00045	0.12344	0.25492
F2RL1	-0.55546	9.65926	-4.88480	0.00047	0.12344	0.20396
CD3E	-0.72224	6.63429	-4.87054	0.00049	0.12344	0.18381
RP4-680D5.8	0.54570	5.20740	4.86520	0.00049	0.12344	0.17627
ANP32A	-0.55416	6.99631	-4.86204	0.00049	0.12344	0.17179
SNX21	0.52104	5.31034	4.83205	0.00052	0.12344	0.12929
SGK1	0.96713	10.71686	4.78829	0.00055	0.12521	0.06698
PKIB	1.21615	7.42352	4.67981	0.00066	0.13426	-0.08885
PIWIL2	-0.63021	6.53883	-4.67694	0.00066	0.13426	-0.09301

CREB3L3	-0.60514	8.90620	-4.63451	0.00071	0.13779	-0.15452
COPA	-0.52042	5.95733	-4.63249	0.00071	0.13779	-0.15745
SLITRK6	0.53496	6.79466	4.59100	0.00076	0.13938	-0.21788
SRSF6	0.87669	7.47103	4.58358	0.00077	0.13938	-0.22872
LOC102724870	0.75350	5.45317	4.58089	0.00078	0.13938	-0.23266
SND1-IT1	-0.58568	6.11336	-4.58067	0.00078	0.13938	-0.23298
RP11-158G18.1	-0.75453	4.65899	-4.55474	0.00081	0.13938	-0.27094
GABARAPL1	-0.53520	6.62988	-4.55370	0.00081	0.13938	-0.27247
ATOH1	-0.54053	5.23870	-4.48685	0.00091	0.14770	-0.37085
RP11-452L6.1	0.53126	4.12645	4.48638	0.00091	0.14770	-0.37154
CXorf56	0.57083	3.62325	4.47938	0.00092	0.14831	-0.38189
SGK494	0.54757	4.88465	4.39676	0.00105	0.15592	-0.50454
MGEA5	-0.60228	6.93627	-4.34932	0.00114	0.15592	-0.57545
ZBED6	0.74142	4.50281	4.34424	0.00115	0.15592	-0.58306
AQP3	-0.51712	8.90604	-4.29105	0.00126	0.15819	-0.66303
DHTKD1	0.65964	6.30531	4.28126	0.00128	0.15958	-0.67779
C7orf73	0.56606	4.51488	4.27899	0.00128	0.15958	-0.68120
CTC-444N24.11	0.51119	6.14780	4.25429	0.00134	0.16107	-0.71852
DGAT2	-0.98445	8.70030	-4.21508	0.00143	0.16450	-0.77795
VPS9D1	0.59123	5.06055	4.20600	0.00145	0.16529	-0.79175
CREBRF	-0.53587	6.93478	-4.18026	0.00151	0.16736	-0.83091
MMP28	0.55265	5.85623	4.11564	0.00169	0.17072	-0.92965
SLC44A4	-0.70026	8.87812	-4.10375	0.00173	0.17072	-0.94788
ADAT1	0.52222	5.95196	4.10358	0.00173	0.17072	-0.94815
STT3A	0.62439	7.94261	4.06050	0.00186	0.17072	-1.01437
NBR1	-0.53231	7.50507	-4.04549	0.00191	0.17072	-1.03749
COL9A3	0.55029	4.97548	4.00837	0.00203	0.17072	-1.09483
AMIGO1	0.94394	4.66458	4.00432	0.00204	0.17072	-1.10111
THNSL2	0.86163	5.37600	4.00256	0.00205	0.17072	-1.10383
EPHX1	-1.05872	9.08978	-3.97670	0.00214	0.17072	-1.14390
SEC61A1	-0.50434	7.99024	-3.97137	0.00216	0.17072	-1.15217
ZNF329	0.67648	5.10214	3.96625	0.00218	0.17072	-1.16012
DHX9	-0.51557	5.93846	-3.96397	0.00219	0.17072	-1.16365
MYL12A	-0.57959	6.08498	-3.93182	0.00232	0.17504	-1.21364
PROM1	-0.76674	7.52792	-3.92538	0.00234	0.17504	-1.22367
LOC100129406	-0.61152	5.16781	-3.85014	0.00267	0.18141	-1.34123
TRIM31	-0.85028	6.40813	-3.84673	0.00268	0.18141	-1.34658
FUT8	0.58390	6.97976	3.83990	0.00272	0.18141	-1.35728
ACOT4	0.80429	6.81690	3.83406	0.00274	0.18141	-1.36645
TOMM40L	0.53614	6.82597	3.82851	0.00277	0.18141	-1.37515
SLC19A3	-0.72907	6.58830	-3.82388	0.00279	0.18141	-1.38242
C1S	-0.52129	7.62577	-3.80916	0.00286	0.18141	-1.40553

COG3	-0.59480	5.58057	-3.79228	0.00295	0.18141	-1.43209
FOXJ3	-0.56968	6.62963	-3.78890	0.00297	0.18141	-1.43740
RP11-288H12.4	0.54866	3.54730	3.77202	0.00306	0.18141	-1.46398
KLRC3	-0.80274	4.61318	-3.76404	0.00310	0.18141	-1.47657
GLUD1	0.51171	9.36073	3.73969	0.00323	0.18252	-1.51499
KLRC4	-0.77720	2.72317	-3.73896	0.00324	0.18252	-1.51614
PKN1	-0.57675	4.64328	-3.71875	0.00335	0.18616	-1.54809
TTC37	-0.54301	6.84924	-3.68732	0.00354	0.19128	-1.59785
ATL2	-0.60541	6.98978	-3.66560	0.00368	0.19528	-1.63229
LINC00969	0.64107	6.92639	3.64878	0.00379	0.19723	-1.65900
TRBC1	-0.56994	8.95521	-3.63409	0.00389	0.19891	-1.68235
KANSL1-AS1	0.96219	4.63764	3.63153	0.00391	0.19891	-1.68643
CRTAM	-0.57713	4.64353	-3.60271	0.00411	0.20175	-1.73229
PRMT6	0.69061	5.45987	3.59877	0.00414	0.20192	-1.73857
GTF2I	-0.67519	5.59509	-3.57531	0.00431	0.20366	-1.77598
MST1R	0.61726	7.66549	3.56038	0.00443	0.20366	-1.79980
ACVR1C	-0.61193	6.51426	-3.55383	0.00448	0.20366	-1.81027
ACKR4	0.60137	6.53840	3.55039	0.00450	0.20366	-1.81576
PHF5A	-0.69443	7.19796	-3.53940	0.00459	0.20493	-1.83333
NUMA1	-0.65188	5.50046	-3.53905	0.00460	0.20493	-1.83389
RP11-274H2.5	-1.09494	4.49793	-3.52047	0.00475	0.20826	-1.86360
AGPAT2	-0.56819	8.66366	-3.51172	0.00482	0.20889	-1.87761
LOC149703	1.18417	8.53230	3.49695	0.00495	0.21101	-1.90126
GPRIN3	-0.61497	5.17014	-3.48193	0.00508	0.21160	-1.92534
RRN3P2	-0.73002	5.06720	-3.47450	0.00515	0.21160	-1.93724
EVA1C	0.51154	4.31605	3.47230	0.00517	0.21160	-1.94078
HSD11B2	0.75785	7.58456	3.47004	0.00519	0.21160	-1.94440
TNIP3	-0.54069	3.00028	-3.45359	0.00534	0.21160	-1.97079
EP400	0.60372	5.38421	3.44272	0.00545	0.21215	-1.98824
CTNNB1	0.51525	7.70904	3.43043	0.00557	0.21419	-2.00799
CCL24	-0.62878	6.12127	-3.42438	0.00563	0.21419	-2.01772
LMAN1	-0.57448	8.67983	-3.41519	0.00572	0.21419	-2.03249
ADCK3	-0.69834	5.64496	-3.40958	0.00578	0.21428	-2.04151
OSTF1	0.54470	7.14226	3.40373	0.00584	0.21428	-2.05093
LOC100505874	-0.67089	4.48811	-3.39114	0.00597	0.21610	-2.07119
C1orf220	0.58330	4.22532	3.36920	0.00621	0.21739	-2.10651
PAQR8	0.78552	8.31307	3.36550	0.00625	0.21764	-2.11248
SLC40A1	-0.57951	9.46765	-3.35055	0.00642	0.21946	-2.13657
LGALS3	0.61221	9.58892	3.33031	0.00665	0.22169	-2.16921
TLCD2	0.59602	7.05199	3.27412	0.00735	0.22805	-2.25992
C1QA	-0.59350	8.26543	-3.26039	0.00753	0.22881	-2.28212
EPHB6	0.52191	4.91749	3.20281	0.00835	0.23786	-2.37526

KCNK10	0.53771	5.55758	3.18877	0.00856	0.23888	-2.39799
WDR43	0.51283	5.77008	3.18811	0.00857	0.23888	-2.39906
SLC23A1	-0.58846	8.27901	-3.18353	0.00864	0.23915	-2.40647
PAQR7	0.76835	6.94455	3.17159	0.00883	0.24073	-2.42581
ITLN1	-1.22274	9.03936	-3.16616	0.00891	0.24209	-2.43460
CD7	-0.51504	5.58705	-3.16084	0.00900	0.24209	-2.44323
RNF19B	-0.58291	7.98615	-3.13187	0.00948	0.24546	-2.49017
KIFAP3	0.59009	7.86259	3.12930	0.00952	0.24560	-2.49434
MRPL2	-0.54724	6.13453	-3.09942	0.01004	0.24742	-2.54276
PRO2852	-0.53699	7.54196	-3.08018	0.01039	0.24982	-2.57395
GLB1L	0.50956	5.81709	3.04362	0.01110	0.25623	-2.63324
REG1B	1.14149	6.70059	3.03410	0.01129	0.25832	-2.64867
ABCD3	-0.52272	8.91800	-3.02763	0.01142	0.25842	-2.65917
LOC100506922	0.86548	5.34618	3.01891	0.01160	0.25868	-2.67331
APOB	-0.57574	11.60567	-3.01593	0.01166	0.25902	-2.67814
TUBB2A	0.55088	9.80511	2.92195	0.01380	0.26990	-2.83049
CTB-50L17.7	-0.68929	5.24425	-2.92088	0.01383	0.26990	-2.83221
LINC00955	-1.16211	7.12160	-2.91902	0.01388	0.26990	-2.83523
LINC00319	0.56075	4.48673	2.91741	0.01392	0.26990	-2.83783
PDGFA	0.51670	6.51385	2.90959	0.01411	0.27035	-2.85051
CHKA	-0.54645	8.19135	-2.90069	0.01434	0.27284	-2.86493
RP11-469M7.1	0.54283	5.68778	2.88375	0.01478	0.27595	-2.89236
CRCT1	0.73522	3.25396	2.88079	0.01486	0.27643	-2.89715
ZNF232	0.52204	6.46894	2.87082	0.01513	0.27732	-2.91329
CISD1	0.51961	9.65943	2.85351	0.01561	0.28139	-2.94131
AP000253.1	0.63798	3.23282	2.84843	0.01575	0.28210	-2.94953
ACOT12	0.93650	3.87274	2.84175	0.01594	0.28391	-2.96033
LOC101929549	-0.51080	5.30434	-2.83179	0.01623	0.28391	-2.97645
FCER1A	-0.58985	4.00506	-2.77707	0.01790	0.29203	-3.06487
EMILIN3	-0.74441	7.59194	-2.76614	0.01825	0.29267	-3.08251
SEC22B	-0.57773	9.50616	-2.75956	0.01847	0.29352	-3.09312
FDPSP2	-0.52551	4.97671	-2.75918	0.01848	0.29352	-3.09373
LINC00339	0.64571	5.12900	2.75429	0.01865	0.29352	-3.10163
BC022892	-0.79279	4.87869	-2.75058	0.01877	0.29352	-3.10762
STAP2	0.55351	8.40924	2.73685	0.01924	0.29726	-3.12975
CTHRC1	0.53036	5.09150	2.72910	0.01951	0.29827	-3.14224
C8orf49	0.57169	4.33879	2.72802	0.01955	0.29827	-3.14397
MIA2	-0.60435	6.33918	-2.69591	0.02070	0.30415	-3.19568
CXCL13	-0.79333	3.67210	-2.68164	0.02124	0.30534	-3.21862
MMP9	-0.85055	5.00860	-2.67841	0.02136	0.30554	-3.22382
USP32P2	0.55726	6.98016	2.66615	0.02184	0.30808	-3.24351
CYP1A1	-1.31651	7.81949	-2.65071	0.02245	0.31059	-3.26831

ELOVL6	0.52659	6.44470	2.64159	0.02282	0.31175	-3.28294
SLC6A8	-0.55991	8.35489	-2.62679	0.02343	0.31371	-3.30667
ESPL1	-1.33881	7.68116	-2.61555	0.02391	0.31563	-3.32467
LOC102723845	0.86563	5.99605	2.60091	0.02454	0.31858	-3.34810
RP11-116O18.1	1.09645	4.03761	2.59970	0.02459	0.31858	-3.35005
LOC101927482	-0.90772	3.71694	-2.59180	0.02494	0.31903	-3.36268
IGK	-1.10821	8.06446	-2.57828	0.02555	0.32073	-3.38430
HRCT1	0.65891	6.81920	2.56027	0.02639	0.32105	-3.41304
C6orf141	0.83092	4.12952	2.54867	0.02694	0.32200	-3.43155
KL	0.57869	5.28451	2.52536	0.02808	0.32591	-3.46867
GSTT1	-1.93318	6.01949	-2.51494	0.02861	0.32752	-3.48523
C4BPB	0.50394	5.15824	2.51066	0.02883	0.32752	-3.49205
C9orf152	-0.64400	6.98236	-2.49301	0.02975	0.32903	-3.52008
LOC729083	-1.38737	5.23576	-2.48720	0.03006	0.33006	-3.52928
PXDN	0.55328	6.87141	2.46132	0.03148	0.33528	-3.57028
SIGLEC15	0.71634	8.69791	2.43941	0.03273	0.33991	-3.60492
CRIP1	0.79176	9.88999	2.42746	0.03343	0.34279	-3.62378
RP11-403P17.3	-0.52237	6.22002	-2.40761	0.03463	0.34568	-3.65505
SPINK4	-0.70098	8.97182	-2.37920	0.03642	0.34999	-3.69970
PTPRD	-0.66629	5.42293	-2.36154	0.03758	0.35438	-3.72737
LOC102724689	0.60863	3.64772	2.35820	0.03780	0.35476	-3.73261
ACCS	0.50370	6.80202	2.35232	0.03819	0.35566	-3.74180
VMP1	-0.50600	6.05597	-2.34586	0.03863	0.35633	-3.75190
RHOBTB1	-0.64566	6.33672	-2.33116	0.03965	0.35923	-3.77486
SERTAD3	0.61468	5.87939	2.32393	0.04016	0.36055	-3.78614
ENDOD1	0.52287	6.59128	2.31381	0.04088	0.36252	-3.80190
DNAJB7	-0.55238	3.82542	-2.29835	0.04201	0.36687	-3.82595
HMOX1	-0.59791	8.26526	-2.26987	0.04417	0.37133	-3.87012
KLRB1	-0.52634	8.45750	-2.25384	0.04544	0.37428	-3.89490
C17orf97	-0.75196	6.02044	-2.23941	0.04660	0.37823	-3.91717
ITGA4	-0.53793	5.24921	-2.22574	0.04773	0.38156	-3.93821
ANXA3	0.93095	6.62290	2.22053	0.04817	0.38246	-3.94623

Table S6. The 106 key targets.

No.	Target	No.	Target
1	TDP1	54	BRAF
2	NFKB1	55	ACE
3	CTSD	56	BACE1
4	TLR4	57	TBXA2R
5	PLAT	58	CHEK1
6	NFE2L2	59	HDAC9

7	HSP90AA1	60	HRH3
8	CHRM3	61	ENPP1
9	DNMT1	62	NR3C1
10	SLC9A1	63	PTK2B
11	NR3C2	64	MTOR
12	ABCC1	65	BCL2
13	PDGFRA	66	TRPM8
14	STAT1	67	TRPA1
15	F13A1	68	CHKA
16	MIF	69	POLH
17	CYP3A4	70	POLI
18	CFTR	71	SMAD3
19	RXFP1	72	AKR1B1
20	GRB2	73	MET
21	LMNA	74	KDR
22	MDM4	75	ACHE
23	NR4A1	76	ESR2
24	LGALS3	77	CXCR4
25	IRAK1	78	SLC1A1
26	TBXAS1	79	SMN1
27	AKR1C2	80	SLC40A1
28	EPHB2	81	HDAC7
29	ACVR1B	82	ADORA2B
30	ADRB1	83	ANPEP
31	PTGS2	84	EPHX1
32	NOS2	85	ALK
33	ZAP70	86	CTSB
34	F2R	87	ALOX5
35	TLR8	88	F7
36	ACVRL1	89	TERT
37	AKR1C1	90	CTSK
38	GPBAR1	91	PLG
39	FCGRT	92	MMP7
40	CETP	93	VCP
41	ITK	94	MMP9
42	P2RX7	95	CCR5
43	LYN	96	F11
44	F10	97	CISD1
45	STAT3	98	TBK1
46	CPT1B	99	GSK3B
47	FLT1	100	MAPKAPK2
48	OPRM1	101	MME

49	ROCK1	102	CDK7
50	GRM5	103	CDK1
51	IL23R	104	ACVR1
52	CASP8	105	ABCG2
53	PROC	106	NPSR1

Table S7. The details of the PPI network.

No.	Target	Node	No.	Target	Node
1	HSP90AA1	88	52	ACHE	14
2	STAT3	78	53	ALOX5	14
3	MMP9	66	54	BACE1	14
4	MTOR	60	55	BRAF	14
5	PTGS2	60	56	F7	14
6	TLR4	58	57	LMNA	14
7	STAT1	48	58	PROC	14
8	CXCR4	46	59	ACVR1	12
9	GSK3B	44	60	ANPEP	12
10	SMAD3	44	61	MDM4	12
11	KDR	42	62	MIF	12
12	ACE	40	63	ACVRL1	10
13	CASP8	40	64	AKR1C1	10
14	GRB2	40	65	CTSK	10
15	NFKB1	34	66	HDAC7	10
16	PLG	34	67	NR3C2	10
17	LYN	32	68	OPRM1	10
18	MET	32	69	ACVR1B	8
19	CDK1	28	70	ADORA2B	8
20	MMP7	28	71	EPHB2	8
21	CTSB	26	72	EPHX1	8
22	TERT	26	73	IL23R	8
23	CFTR	24	74	P2RX7	8
24	NR3C1	24	75	POLH	8
25	CHEK1	22	76	POLI	8
26	CTSD	22	77	TBXA2R	8
27	PTK2B	22	78	TBXAS1	8
28	TBK1	22	79	TDP1	8
29	ALK	20	80	CHKA	6
30	CYP3A4	20	81	F11	6
31	DNMT1	20	82	F13A1	6
32	ESR2	20	83	HDAC9	6
33	FLT1	20	84	HRH3	6
34	MME	20	85	ITK	6
35	NFE2L2	20	86	MAPKAPK2	6
36	ZAP70	20	87	SLC9A1	6

37	BCL2	18	88	TRPA1	6
38	F10	18	89	ABCC1	4
39	IRAK1	18	90	ADRB1	4
40	LGALS3	18	91	AKR1B1	4
41	PLAT	18	92	AKR1C2	4
42	VCP	18	93	CDK7	4
43	ABCG2	16	94	SMN1	4
44	CCR5	16	95	TRPM8	4
45	F2R	16	96	CETP	2
46	GRM5	16	97	CHRM3	2
47	NOS2	16	98	CISD1	2
48	NR4A1	16	99	ENPP1	2
49	PDGFRA	16	100	GPBAR1	2
50	ROCK1	16	101	NPSR1	2
51	TLR8	16	102	SLC1A1	2

Table S8. The details of the GO. (the top 20 terms of BP, CC and MF)

ONTOLOGY	ID	Description	GeneRatio	BgRatio	pvalue	p.adjust	qvalue	geneID	Count
BP	GO:0061041	regulation of wound healing	14/106	137/18903	3.36E-14	5.11E-11	2.87E-11	PLAT/NFE2L2/PDGFRA/EPHB2/F2R/PROC/TBXA2R/MTOR/SMAD3/CXCR4/ALOX5/F7/PLG/F11	14
BP	GO:1903034	regulation of response to wounding	15/106	172/18903	4.14E-14	5.11E-11	2.87E-11	PLAT/NFE2L2/PDGFRA/EPHB2/F2R/PROC/BRAF/TBXA2R/MTOR/SMAD3/CXCR4/ALOX5/F7/PLG/F11	15
BP	GO:0042060	wound healing	21/106	442/18903	4.45E-14	5.11E-11	2.87E-11	TLR4/PLAT/NFE2L2/PDGFRA/F13A1/EPHB2/F2R/ACVRL1/LYN/F10/OPRM1/PROC/TBXA2R/MTOR/SMAD3/KDR/CXCR4/ALOX5/F7/PLG/F11	21
BP	GO:0036293	response to decreased oxygen levels	18/106	309/18903	1.12E-13	9.62E-11	5.40E-11	PLAT/NFE2L2/SLC9A1/LMNA/MDM4/IRAK1/PTGS2/NOS2/ACVRL1/ACE/PTK2B/MTOR/BCL2/SMAD3/CXCR4/SLC1A1/F7/TERT	18
BP	GO:0009410	response to xenobiotic stimulus	20/106	432/18903	3.12E-13	2.15E-10	1.21E-10	NFE2L2/HSP90AA1/ABCC1/STAT1/CYP3A4/PTGS2/NOS2/AKR1C1/P2RX7/LYN/BRAF/ACE/TBXA2R/PTK2B/BCL2/TRPA1/CXCR4/SLC1A1/EPHX1/CDK1	20

BP	GO: 0070 482	response to oxygen levels	18/106	337/1890 3	4.91E-13	2.67E-10	1.50E-10	PLAT/NFE2L2/SLC9A1/L MNA/MDM4/IRAK1/PTG S2/NOS2/ACVRL1/ACE/ PTK2B/MTOR/BCL2/SM AD3/CXCR4/SLC1A1/F7/ TERT	18
BP	GO: 0032 496	response to lipopolysac charide	18/106	339/1890 3	5.42E-13	2.67E-10	1.50E-10	NFKB1/TLR4/MIF/NR4A 1/IRAK1/EPHB2/PTGS2/ NOS2/F2R/P2RX7/LYN/ OPRM1/IL23R/CASP8/A CE/TBXA2R/CCR5/MAP KAPK2	18
BP	GO: 0001 666	response to hypoxia	17/106	296/1890 3	7.21E-13	3.10E-10	1.74E-10	PLAT/NFE2L2/SLC9A1/L MNA/MDM4/IRAK1/PTG S2/NOS2/ACVRL1/ACE/ PTK2B/MTOR/BCL2/SM AD3/CXCR4/F7/TERT	17
BP	GO: 0032 103	positive regulation of response to external stimulus	20/106	464/1890 3	1.17E-12	4.46E-10	2.50E-10	TLR4/PLAT/HSP90AA1/ ABCC1/MIF/PTGS2/TLR 8/LYN/OPRM1/BRAF/AC E/PTK2B/SMAD3/MET/K DR/CXCR4/ADORA2B/F 7/PLG/TBK1	20
BP	GO: 0002 237	response to molecule of bacterial origin	18/106	360/1890 3	1.50E-12	5.15E-10	2.89E-10	NFKB1/TLR4/MIF/NR4A 1/IRAK1/EPHB2/PTGS2/ NOS2/F2R/P2RX7/LYN/ OPRM1/IL23R/CASP8/A CE/TBXA2R/CCR5/MAP KAPK2	18
BP	GO: 0009 266	response to temperature stimulus	14/106	186/1890 3	2.34E-12	7.32E-10	4.11E-10	HSP90AA1/SLC9A1/IRA K1/ADRB1/PTGS2/LYN/ CASP8/MTOR/TRPM8/T RPA1/CXCR4/VCP/GSK3 B/MAPKAPK2	14
BP	GO: 1901 653	cellular response to peptide	18/106	373/1890 3	2.71E-12	7.79E-10	4.37E-10	NFKB1/TLR4/PLAT/NFE 2L2/SLC9A1/ABCC1/ST AT1/GRB2/NR4A1/EPHB 2/LYN/STAT3/ROCK1/G RM5/BACE1/HDAC9/EN PP1/GSK3B	18
BP	GO: 0050 878	regulation of body fluid levels	18/106	390/1890 3	5.72E-12	1.47E-09	8.25E-10	TLR4/PLAT/NFE2L2/CH RM3/PDGFRA/F13A1/CF TR/EPHB2/F2R/LYN/F10 /PROC/TBXA2R/AKR1B 1/MET/F7/PLG/F11	18
BP	GO: 0030 193	regulation of blood coagulation	10/106	70/18903	5.98E-12	1.47E-09	8.25E-10	PLAT/NFE2L2/PDGFRA/ EPHB2/F2R/PROC/TBXA 2R/F7/PLG/F11	10
BP	GO: 1900 046	regulation of hemostasis	10/106	72/18903	8.00E-12	1.84E-09	1.03E-09	PLAT/NFE2L2/PDGFRA/ EPHB2/F2R/PROC/TBXA 2R/F7/PLG/F11	10

BP	GO:0050818	regulation of coagulation	10/106	75/18903	1.22E-11	2.62E-09	1.47E-09	PLAT/NFE2L2/PDGFRA/EPHB2/F2R/PROC/TBXA2R/F7/PLG/F11	10
BP	GO:0001819	positive regulation of cytokine production	19/106	486/18903	2.48E-11	5.01E-09	2.82E-09	TLR4/HSP90AA1/STAT1/MIF/IRAK1/EPHB2/PTGS2/NOS2/F2R/TLR8/ITK/P2RX7/STAT3/IL23R/CASP8/SMAD3/ADORA2B/TBK1/MAPKAPK2	19
BP	GO:0007596	blood coagulation	14/106	225/18903	3.07E-11	5.87E-09	3.30E-09	TLR4/PLAT/NFE2L2/PDGFRA/F13A1/EPHB2/F2R/LYN/F10/PROC/TBXA2R/F7/PLG/F11	14
BP	GO:0046777	protein autophosphorylation	14/106	226/18903	3.26E-11	5.90E-09	3.31E-09	PDGFRA/IRAK1/ACVR1B/ZAP70/LYN/FLT1/ACE/ENPP1/PTK2B/MTOR/KDR/ALK/GSK3B/MAPKAPK2	14
BP	GO:0018108	peptidyl-tyrosine phosphorylation	17/106	380/18903	3.83E-11	6.51E-09	3.65E-09	PDGFRA/MIF/EPHB2/ZAP70/ITK/LYN/FLT1/GRM5/IL23R/ACE/PTK2B/MTOR/CHKA/MET/KDR/ALK/ACVR1	17
CC	GO:0045121	membrane raft	15/106	326/19869	2.06E-10	2.86E-08	2.19E-08	CTSD/SLC9A1/PTGS2/ZAP70/F2R/LYN/OPRM1/CASP8/BACE1/PTK2B/TRPM8/KDR/SLC1A1/MME/ABCG2	15
CC	GO:0098857	membrane microdomain	15/106	327/19869	2.15E-10	2.86E-08	2.19E-08	CTSD/SLC9A1/PTGS2/ZAP70/F2R/LYN/OPRM1/CASP8/BACE1/PTK2B/TRPM8/KDR/SLC1A1/MME/ABCG2	15
CC	GO:0009897	external side of plasma membrane	17/106	462/19869	3.70E-10	3.28E-08	2.52E-08	TLR4/PDGFRA/TLR8/FCGRT/P2RX7/F10/IL23R/ACE/TRPM8/KDR/CXCR4/ANPEP/CTSB/CTSK/PLG/CCR5/ABCG2	17
CC	GO:1904813	ficolin-1-rich granule lumen	7/106	124/19869	4.54E-06	0.000302166	0.000231976	CTSD/HSP90AA1/MIF/CTSB/ALOX5/VCP/MMP9	7
CC	GO:0101002	ficolin-1-rich granule	8/106	185/19869	6.73E-06	0.000357879	0.000274746	CTSD/HSP90AA1/MIF/LGALS3/CTSB/ALOX5/VCP/MMP9	8
CC	GO:0060205	cytoplasmic vesicle lumen	10/106	325/19869	9.49E-06	0.000380494	0.000292108	NFKB1/CTSD/HSP90AA1/F13A1/MIF/ROCK1/BACE1/ALOX5/PLG/VCP	10

CC	GO: 0031 983	vesicle lumen	10/106	327/1986 9	1.00E-05	0.00038049 4	0.00029210 8	NFKB1/CTSD/HSP90AA 1/F13A1/MIF/ROCK1/BA CE1/ALOX5/PLG/VCP	10
CC	GO: 0098 685	Schaffer collateral - CA1 synapse	5/106	72/19869	4.14E-05	0.00137638 7	0.00105666 4	PLAT/ADRB1/GRM5/AD ORA2B/PLG	5
CC	GO: 0034 774	secretory granule lumen	9/106	322/1986 9	5.71E-05	0.00158342	0.00121560 5	NFKB1/CTSD/HSP90AA 1/F13A1/MIF/ROCK1/AL OX5/PLG/VCP	9
CC	GO: 0098 978	glutamaterg ic synapse	9/106	324/1986 9	5.99E-05	0.00158342	0.00121560 5	PLAT/EPHB2/LYN/GRM 5/PTK2B/ADORA2B/PLG /VCP/GSK3B	9
CC	GO: 0009 925	basal plasma membrane	8/106	254/1986 9	6.55E-05	0.00158342	0.00121560 5	HSP90AA1/CHRM3/SLC 9A1/ABCC1/ACE/ENPP1/ MET/SLC40A1	8
CC	GO: 0045 178	basal part of cell	8/106	272/1986 9	0.00010543 9	0.00228647 4	0.00175534 6	HSP90AA1/CHRM3/SLC 9A1/ABCC1/ACE/ENPP1/ MET/SLC40A1	8
CC	GO: 0045 177	apical part of cell	10/106	435/1986 9	0.00011174 5	0.00228647 4	0.00175534 6	PLAT/HSP90AA1/SLC9A 1/ABCC1/CFTR/SLC1A1/ CTSB/CTSK/ACVR1/AB CG2	10
CC	GO: 0043 025	neuronal cell body	10/106	497/1986 9	0.00032650 1	0.00620351 8	0.00476249 5	HSP90AA1/EPHB2/ACV RL1/P2RX7/OPRM1/BAC E1/PTK2B/SLC1A1/SMN 1/MME	10
CC	GO: 0005 769	early endosome	9/106	414/1986 9	0.00037528 4	0.00665504 4	0.00510913 6	TLR4/CFTR/ADRB1/F2R/ BACE1/KDR/CXCR4/SL C1A1/MME	9
CC	GO: 0036 019	endolysoso me	3/106	29/19869	0.00048748 7	0.00767005 5	0.00588836 9	TLR8/CTSB/CTSK	3
CC	GO: 1902 554	serine/threo nine protein kinase complex	5/106	122/1986 9	0.00049678 5	0.00767005 5	0.00588836 9	ACVR1B/TBK1/CDK7/C DK1/ACVR1	5
CC	GO: 0062 023	collagen-containing extracellula r matrix	9/106	433/1986 9	0.00051902 6	0.00767005 5	0.00588836 9	CTSD/HSP90AA1/F13A1/ LGALS3/ACHE/CTSB/F7 /PLG/MMP9	9
CC	GO: 0016 324	apical plasma membrane	8/106	368/1986 9	0.00079957 3	0.01081749 9	0.00830468 9	HSP90AA1/SLC9A1/ABC C1/CFTR/SLC1A1/CTSB/ CTSK/ABCG2	8

CC	GO: 1902 911	protein kinase complex	5/106	136/1986 9	0.00081334 6	0.01081749 9	0.00830468 9	ACVR1B/TBK1/CDK7/C DK1/ACVR1	5
MF	GO: 0004 713	protein tyrosine kinase activity	11/106	138/1843 2	4.20E-10	1.67E-07	1.14E-07	PDGFRA/EPHB2/ZAP70/ ITK/LYN/FLT1/PTK2B/C HKA/MET/KDR/ALK	11
MF	GO: 0019 199	transmembr ane receptor protein kinase activity	9/106	79/18432	7.18E-10	1.67E-07	1.14E-07	PDGFRA/EPHB2/ACVR1 B/ACVRL1/FLT1/MET/K DR/ALK/ACVR1	9
MF	GO: 0019 903	protein phosphatas e binding	10/106	149/1843 2	1.44E-08	1.60E-06	1.08E-06	HSP90AA1/SLC9A1/STA T1/GRB2/LGALS3/STAT 3/BCL2/MET/VCP/TBK1	10
MF	GO: 0019 902	phosphatas e binding	11/106	195/1843 2	1.62E-08	1.60E-06	1.08E-06	HSP90AA1/SLC9A1/STA T1/GRB2/LGALS3/STAT 3/BCL2/SMAD3/MET/VC P/TBK1	11
MF	GO: 0017 171	serine hydrolase activity	11/106	196/1843 2	1.71E-08	1.60E-06	1.08E-06	PLAT/F10/PROC/ACE/A CHE/F7/CTSK/PLG/MMP 7/MMP9/F11	11
MF	GO: 0004 175	endopeptid ase activity	15/106	426/1843 2	2.14E-08	1.66E-06	1.13E-06	CTSD/PLAT/F10/CASP8/ PROC/ACE/BACE1/CTS B/F7/CTSK/PLG/MMP7/ MMP9/F11/MME	15
MF	GO: 0008 236	serine-type peptidase activity	10/106	192/1843 2	1.59E-07	1.00E-05	6.80E-06	PLAT/F10/PROC/ACE/F7 /CTSK/PLG/MMP7/MMP 9/F11	10
MF	GO: 0004 674	protein serine/threo nine kinase activity	14/106	430/1843 2	1.72E-07	1.00E-05	6.80E-06	IRAK1/ACVR1B/ACVRL 1/ROCK1/BRAF/CHEK1/ PTK2B/MTOR/TBK1/GS K3B/MAPKAPK2/CDK7/ CDK1/ACVR1	14
MF	GO: 0004 252	serine-type endopeptid ase activity	9/106	174/1843 2	7.25E-07	3.75E-05	2.55E-05	PLAT/F10/PROC/F7/CTS K/PLG/MMP7/MMP9/F11	9
MF	GO: 0004 714	transmembr ane receptor protein tyrosine kinase activity	6/106	60/18432	1.22E-06	5.69E-05	3.87E-05	PDGFRA/EPHB2/FLT1/M ET/KDR/ALK	6
MF	GO: 0044 389	ubiquitin-like protein ligase binding	11/106	318/1843 2	2.20E-06	9.30E-05	6.33E-05	HSP90AA1/STAT1/ACV R1B/LYN/CASP8/PTK2B /BCL2/SMAD3/CXCR4/V CP/GSK3B	11

MF	GO: 0031 625	ubiquitin protein ligase binding	10/106	299/1843 2	8.76E-06	0.00034021 5	0.00023131 8	HSP90AA1/ACVR1B/LY N/CASP8/PTK2B/BCL2/S MAD3/CXCR4/VCP/GSK 3B	10
MF	GO: 0004 879	nuclear receptor activity	5/106	52/18432	1.20E-05	0.00039915 1	0.00027139 1	NR3C2/NR4A1/STAT3/N R3C1/ESR2	5
MF	GO: 0098 531	ligand-activated transcriptio n factor activity	5/106	52/18432	1.20E-05	0.00039915 1	0.00027139 1	NR3C2/NR4A1/STAT3/N R3C1/ESR2	5
MF	GO: 0051 219	phosphopro tein binding	6/106	92/18432	1.50E-05	0.00046545 4	0.00031647 1	PLAT/GRB2/ZAP70/LYN /MTOR/TBK1	6
MF	GO: 0017 002	activin receptor activity	3/106	10/18432	2.15E-05	0.00059052 6	0.00040151	ACVR1B/ACVRL1/ACV R1	3
MF	GO: 0099 528	G protein-coupled neurotrans mitter receptor activity	3/106	10/18432	2.15E-05	0.00059052 6	0.00040151	CHRM3/ADRB1/HRH3	3
MF	GO: 0004 032	alditol:NA DP+ 1-oxidoreduct ase activity	3/106	12/18432	3.92E-05	0.00101394 6	0.00068940 1	AKR1C2/AKR1C1/AKR1 B1	3
MF	GO: 0106 310	protein serine kinase activity	10/106	363/1843 2	4.62E-05	0.00111817 7	0.00076027	IRAK1/ROCK1/BRAF/C HEK1/MTOR/TBK1/GSK 3B/MAPKAPK2/CDK7/C DK1	10
MF	GO: 0005 518	collagen binding	5/106	69/18432	4.80E-05	0.00111817 7	0.00076027	SMAD3/ACHE/CTSB/CT SK/MMP9	5

Table S9. The details of the KEGG.

ID	Description	GeneRatio	BgRatio	pvalue	p.adjust	qvalue	geneID	Count
hsa05161	Hepatitis B	13/100	162/8227	6.68E-08	1.57E-05	9.35E-06	NFKB1/TLR4/STAT1/GRB 2/IRAK1/STAT3/CASP8/BR AF/PTK2B/BCL2/SMAD3/ MMP9/TBK1	13
hsa05215	Prostate cancer	10/100	97/8227	2.34E-07	2.75E-05	1.64E-05	NFKB1/PLAT/HSP90AA1/P DGFRA/GRB2/BRAF/MTO R/BCL2/MMP9/GSK3B	10
hsa01521	EGFR tyrosine kinase inhibitor resistance	9/100	79/8227	4.10E-07	3.21E-05	1.91E-05	PDGFRA/GRB2/STAT3/BR AF/MTOR/BCL2/MET/KD R/GSK3B	9
hsa05145	Toxoplasmosis	10/100	112/8227	9.06E-07	4.93E-05	2.94E-05	NFKB1/TLR4/STAT1/IRAK 1/NOS2/STAT3/CASP8/BC L2/ALOX5/CCR5	10

hsa04020	Calcium signaling pathway	14/100	240/8227	1.05E-06	4.93E-05	2.94E-05	CHRM3/PDGFRA/ADRB1/NOS2/F2R/P2RX7/FLT1/GRM5/TBXA2R/PTK2B/MET/KDR/CXCR4/ADORA2B	14
hsa05163	Human cytomegalovirus infection	13/100	225/8227	2.93E-06	0.000108392	6.46E-05	NFKB1/PDGFRA/GRB2/PTGS2/STAT3/ROCK1/CASP8/PTK2B/MTOR/CXCR4/CCR5/TBK1/GSK3B	13
hsa04062	Chemokine signaling pathway	12/100	192/8227	3.23E-06	0.000108392	6.46E-05	NFKB1/STAT1/GRB2/ITK/LYN/STAT3/ROCK1/BRAF/PTK2B/CXCR4/CCR5/GSK3B	12
hsa04610	Complement and coagulation cascades	8/100	86/8227	8.73E-06	0.000234355	0.000139616	PLAT/F13A1/F2R/F10/PROC/F7/PLG/F11	8
hsa05170	Human immunodeficiency virus 1 infection	12/100	212/8227	8.98E-06	0.000234355	0.000139616	NFKB1/TLR4/IRAK1/CASP8/CHEK1/PTK2B/MTOR/BCL2/CXCR4/CCR5/TBK1/CDK1	12
hsa05417	Lipid and atherosclerosis	12/100	215/8227	1.04E-05	0.000243357	0.000144979	NFKB1/TLR4/NFE2L2/HSP90AA1/IRAK1/LYN/STAT3/CASP8/BCL2/MMP9/TBK1/GSK3B	12
hsa05167	Kaposi sarcoma-associated herpesvirus infection	11/100	194/8227	2.14E-05	0.000457982	0.00027284	NFKB1/STAT1/PTGS2/LYN/STAT3/CASP8/MTOR/CCR5/TBK1/GSK3B/MAPKAPK2	11
hsa05203	Viral carcinogenesis	11/100	204/8227	3.42E-05	0.000640317	0.000381465	NFKB1/GRB2/LYN/STAT3/CASP8/CHEK1/HDAC9/HDAC7/CCR5/MAPKAPK2/CDK1	11
hsa04620	Toll-like receptor signaling pathway	8/100	104/8227	3.54E-05	0.000640317	0.000381465	NFKB1/TLR4/STAT1/IRAK1/TLR8/CASP8/CTSK/TBK1	8
hsa05162	Measles	9/100	139/8227	4.48E-05	0.000728778	0.000434166	NFKB1/TLR4/STAT1/IRAK1/STAT3/CASP8/BCL2/TBK1/GSK3B	9
hsa04659	Th17 cell differentiation	8/100	108/8227	4.65E-05	0.000728778	0.000434166	NFKB1/HSP90AA1/STAT1/ZAP70/STAT3/IL23R/MTOR/SMAD3	8
hsa05206	MicroRNAs in cancer	13/100	310/8227	8.90E-05	0.001307668	0.000779036	NFKB1/DNMT1/ABCC1/PDGFRA/GRB2/MDM4/PTGS2/STAT3/ROCK1/MTOR/BCL2/MET/MMP9	13
hsa05235	PD-L1 expression and PD-1 checkpoint pathway in cancer	7/100	89/8227	9.68E-05	0.001338426	0.00079736	NFKB1/TLR4/STAT1/ZAP70/STAT3/MTOR/ALK	7
hsa05321	Inflammatory bowel disease	6/100	65/8227	0.000128346	0.00167563	0.000998248	NFKB1/TLR4/STAT1/STAT3/IL23R/SMAD3	6
hsa04657	IL-17 signaling pathway	7/100	94/8227	0.000136893	0.001693145	0.001008682	NFKB1/HSP90AA1/PTGS2/CASP8/MMP9/TBK1/GSK3B	7
hsa05205	Proteoglycans in cancer	10/100	205/8227	0.000181998	0.002138481	0.001273989	TLR4/SLC9A1/GRB2/STAT3/ROCK1/BRAF/MTOR/MET/KDR/MMP9	10
hsa04917	Prolactin signaling pathway	6/100	70/8227	0.000194012	0.002171083	0.001293411	NFKB1/STAT1/GRB2/STAT3/ESR2/GSK3B	6
hsa05207	Chemical carcinogenesis - receptor activation	10/100	212/8227	0.000239136	0.002519965	0.001501256	NFKB1/HSP90AA1/CYP3A4/GRB2/ADRB1/STAT3/MTOR/BCL2/ESR2/EPHX1	10
hsa05135	Yersinia infection	8/100	137/8227	0.00024739	0.002519965	0.001501256	NFKB1/TLR4/IRAK1/ZAP70/ROCK1/PTK2B/TBK1/GSK3B	8
hsa04064	NF-kappa B signaling pathway	7/100	104/8227	0.000257358	0.002519965	0.001501256	NFKB1/TLR4/IRAK1/PTGS2/ZAP70/LYN/BCL2	7
hsa05418	Fluid shear stress and atherosclerosis	8/100	139/8227	0.000273158	0.002567687	0.001529686	NFKB1/PLAT/NFE2L2/HSP90AA1/BCL2/KDR/MMP9/ACVR1	8
hsa05212	Pancreatic cancer	6/100	76/8227	0.000305067	0.002757335	0.001642668	NFKB1/STAT1/STAT3/BRAF/MTOR/SMAD3	6
hsa05140	Leishmaniasis	6/100	77/8227	0.000327631	0.002784185	0.001658664	NFKB1/TLR4/STAT1/IRAK1/PTGS2/NOS2	6

hsa04151	PI3K-Akt signaling pathway	13/100	354/8227	0.000331896	0.002784185	0.001658664	NFKB1/TLR4/HSP90AA1/PDGFRA/GRB2/NR4A1/F2R/FLT1/MTOR/BCL2/MET/KDR/GSK3B	13
hsa04066	HIF-1 signaling pathway	7/100	109/8227	0.00034358	0.002784185	0.001658664	NFKB1/TLR4/NOS2/STAT3/FLT1/MTOR/BCL2	7
hsa04621	NOD-like receptor signaling pathway	9/100	186/8227	0.000411672	0.003224762	0.001921135	NFKB1/TLR4/HSP90AA1/STAT1/P2RX7/CASP8/BCL2/CTSB/TBK1	9
hsa05226	Gastric cancer	8/100	149/8227	0.000437169	0.003314026	0.001974314	GRB2/BRAF/MTOR/BCL2/SMAD3/MET/TERT/GSK3B	8
hsa04080	Neuroactive ligand-receptor interaction	13/100	367/8227	0.000469209	0.003445752	0.002052788	CHRM3/RXFP1/ADRB1/F2R/P2RX7/OPRM1/GRM5/TBXA2R/HRH3/NR3C1/ADORA2B/PLG/NPSR1	13
hsa04722	Neurotrophin signaling pathway	7/100	119/8227	0.000585194	0.004112846	0.002450206	NFKB1/GRB2/IRAK1/BRAF/BCL2/GSK3B/MAPKAPK2	7
hsa05210	Colorectal cancer	6/100	86/8227	0.00059505	0.004112846	0.002450206	GRB2/BRAF/MTOR/BCL2/SMAD3/GSK3B	6
hsa05160	Hepatitis C	8/100	157/8227	0.000619582	0.00416005	0.002478328	NFKB1/STAT1/GRB2/STAT3/CASP8/BRAF/TBK1/GSK3B	8
hsa04510	Focal adhesion	9/100	203/8227	0.000775998	0.005065541	0.003017769	PDGFRA/GRB2/FLT1/ROCK1/BRAF/BCL2/MET/KDR/GSK3B	9
hsa05225	Hepatocellular carcinoma	8/100	168/8227	0.000966318	0.006137422	0.003656337	NFE2L2/GRB2/BRAF/MTOR/SMAD3/MET/TERT/GSK3B	8
hsa01522	Endocrine resistance	6/100	98/8227	0.001185194	0.00732949	0.004366505	GRB2/BRAF/MTOR/BCL2/ESR2/MMP9	6
hsa05221	Acute myeloid leukemia	5/100	67/8227	0.001276656	0.007574704	0.00451259	NFKB1/GRB2/STAT3/BRAF/MTOR	5
hsa04210	Apoptosis	7/100	136/8227	0.001289311	0.007574704	0.00451259	NFKB1/CTSD/LMNA/CASP8/BCL2/CTSB/CTSK	7
hsa04915	Estrogen signaling pathway	7/100	138/8227	0.001403413	0.008043955	0.004792144	CTSD/HSP90AA1/GRB2/OPRM1/BCL2/ESR2/MMP9	7
hsa05142	Chagas disease	6/100	102/8227	0.001458241	0.008159208	0.004860805	NFKB1/TLR4/IRAK1/NOS2/CASP8/ACE	6
hsa05152	Tuberculosis	8/100	180/8227	0.001506702	0.008234303	0.004905542	NFKB1/CTSD/TLR4/STAT1/IRAK1/NOS2/CASP8/BCL2	8
hsa04936	Alcoholic liver disease	7/100	142/8227	0.001655247	0.008840523	0.005266695	NFKB1/TLR4/IRAK1/CPT1B/CASP8/TBK1/GSK3B	7
hsa05223	Non-small cell lung cancer	5/100	72/8227	0.001763288	0.00920828	0.005485784	GRB2/STAT3/BRAF/MET/ALK	5
hsa04115	p53 signaling pathway	5/100	73/8227	0.001874955	0.009578575	0.005706385	MDM4/CASP8/CHEK1/BCL2/CDK1	5
hsa05171	Coronavirus disease - COVID-19	9/100	232/8227	0.001976562	0.00988281	0.005887631	NFKB1/TLR4/STAT1/F13A1/IRAK1/TLR8/STAT3/ACE/TBK1	9
hsa04614	Renin-angiotensin system	3/100	23/8227	0.00258648	0.012662974	0.0075439	ACE/ANPEP/MME	3
hsa04010	MAPK signaling pathway	10/100	294/8227	0.002939341	0.01409684	0.008398118	NFKB1/PDGFRA/GRB2/NR4A1/IRAK1/FLT1/BRAF/MET/KDR/MAPKAPK2	10
hsa05169	Epstein-Barr virus infection	8/100	202/8227	0.003100445	0.01457209	0.008681245	NFKB1/STAT1/IRAK1/LYN/STAT3/CASP8/BCL2/TBK1	8
hsa05415	Diabetic cardiomyopathy	8/100	203/8227	0.003195807	0.014725775	0.008772802	NFKB1/CTSD/CPT1B/ACE/MTOR/SMAD3/MMP9/GSK3B	8
hsa04630	JAK-STAT signaling pathway	7/100	166/8227	0.003987136	0.018018786	0.010734596	PDGFRA/STAT1/GRB2/STAT3/IL23R/MTOR/BCL2	7
hsa04540	Gap junction	5/100	88/8227	0.004238862	0.018794955	0.011196994	PDGFRA/GRB2/ADRB1/GRM5/CDK1	5
hsa04976	Bile secretion	5/100	89/8227	0.004448659	0.019359906	0.011533561	SLC9A1/CYP3A4/CFTR/EPHX1/ABCG2	5
hsa05134	Legionellosis	4/100	57/8227	0.004958456	0.021186132	0.012621526	NFKB1/TLR4/CASP8/VCP	4

hsa01523	Antifolate resistance	3/100	30/8227	0.005576031	0.023212497	0.013828722	NFKB1/ABCC1/ABCG2	3
hsa05208	Chemical carcinogenesis - reactive oxygen species	8/100	223/8227	0.005630265	0.023212497	0.013828722	NFKB1/NFE2L2/GRB2/AKR1C2/AKR1C1/BRAF/MET/EPHX1	8
hsa05010	Alzheimer disease	11/100	384/8227	0.006717088	0.027215788	0.016213661	NFKB1/CHRM3/PTGS2/NOS2/GRM5/CASP8/BRAF/BACE1/MTOR/GSK3B/MME	11
hsa04933	AGE-RAGE signaling pathway in diabetic complications	5/100	100/8227	0.007268598	0.028951195	0.01724752	NFKB1/STAT1/STAT3/BCL2/SMAD3	5
hsa04550	Signaling pathways regulating pluripotency of stem cells	6/100	143/8227	0.007770059	0.030224484	0.018006075	GRB2/ACVR1B/STAT3/SMAD3/GSK3B/ACVR1	6
hsa04014	Ras signaling pathway	8/100	236/8227	0.007845504	0.030224484	0.018006075	NFKB1/PDGFRA/GRB2/ZAP70/FLT1/MET/KDR/TBK1	8
hsa04625	C-type lectin receptor signaling pathway	5/100	104/8227	0.008548101	0.031885773	0.018995779	NFKB1/STAT1/PTGS2/CASP8/MAPKAPK2	5
hsa04660	T cell receptor signaling pathway	5/100	104/8227	0.008548101	0.031885773	0.018995779	NFKB1/GRB2/ZAP70/ITK/GSK3B	5
hsa04072	Phospholipase D signaling pathway	6/100	148/8227	0.009132236	0.03353243	0.019976767	PDGFRA/GRB2/F2R/GRM5/PTK2B/MTOR	6
hsa04920	Adipocytokine signaling pathway	4/100	69/8227	0.009716799	0.034597692	0.020611391	NFKB1/STAT3/CPT1B/MTOR	4
hsa05204	Chemical carcinogenesis - DNA adducts	4/100	69/8227	0.009716799	0.034597692	0.020611391	CYP3A4/AKR1C2/PTGS2/EPHX1	4
hsa04931	Insulin resistance	5/100	108/8227	0.009976112	0.034990841	0.020845607	NFKB1/STAT3/CPT1B/MTOR/GSK3B	5
